# Systematic review of literature to evaluate global distribution of species of the *Sporothrix* genus stored in culture collections

**DOI:** 10.3389/fcimb.2024.1382508

**Published:** 2024-05-17

**Authors:** Debora Salgado Morgado, Rodolfo Castro, Marcelo Ribeiro-Alves, Danielly Corrêa-Moreira, Júlio Castro Alves de Lima e Silva, Rodrigo Caldas Menezes, Manoel Marques Evangelista Oliveira

**Affiliations:** ^1^ Laboratory of Taxonomy, Biochemistry and Bioprospecting of Fungi, Oswaldo Cruz Institute, Oswaldo Cruz Foundation, Rio de Janeiro, Brazil; ^2^ Sergio Arouca National School of Public Health, Oswaldo Cruz Foundation, Rio de Janeiro, Brazil; ^3^ Institute of Collective Health, Federal University of the State of Rio de Janeiro, Rio de Janeiro, Brazil; ^4^ Laboratory of AIDS and Molecular Immunology, Oswaldo Cruz Institute, Oswaldo Cruz Foundation, Rio de Janeiro, Brazil; ^5^ Clinical Reseacrh Plataform, Oswaldo Cruz Foundation, Rio de Janeiro, Brazil; ^6^ Laboratory of Clinical Research on Dermatozoonoses in Domestic Animals, Evandro Chagas National Institute of Infectious Diseases, Oswaldo Cruz Foundation, Rio de Janeiro, Brazil

**Keywords:** *Sporothrix* sp, sporotrichosis, culture collections, preservation, polyphasic taxonomy, systematic review

## Abstract

**Introduction:**

Sporotrichosis is a subcutaneous mycosis caused by fungi of the genus *Sporothrix* sp. Phenotypic and genotypic differences have been associated with their geographic distribution, virulence, or clinical manifestation of sporotrichosis. In the past decade, the interest in identifying species of the *Sporothrix* sp. has been increasing, due to its epidemiological importance and, in consequence, is important to know how to preserve them for future studies, in culture collection.

**Aims:**

The purposes of this study were to analyze the global distribution of environmental isolates and/or causal agents of sporotrichosis identified by polyphasic taxonomy, with mandatory use of molecular identification, and to evaluate the percentages and distribution of isolates stored in culture collections.

**Methods:**

A systematic review of articles on animal and human sporotrichosis and/or environmental isolation of the fungus, from 2007 to 2023, was done. Results: Our results demonstrated that, *S. globosa*, *S. schenckii*, and *S. brasiliensis* were the most identified species. With respect to the deposit and maintenance of species, we observed that only 17% of the strains of *Sporothrix* sp. isolated in the world are preserved in a culture collection.

**Conclusions:**

This systematic review confirmed a difficulty in obtaining the frequency of *Sporothrix* species stored in culture collection and insufficient data on the molecular identification mainly of animal sporotrichosis and isolation of *Sporothrix* sp. in environmental samples.

## Introduction

Sporotrichosis is a subcutaneous mycosis caused by thermodimorphic fungi of the *Sporothrix* genus. Molecular studies demonstrated that isolates of *Sporothrix schenckii* could not be considered as a single species but a complex of species causing sporotrichosis (Marimon et al., 2006). Since the variability among species may influence the epidemiology, transmission, clinical aspects, and outcome of the infection, it is important to correctly identify the etiological agent.

According to De Beer et al. (2016), this complex is distributed all over the world and divided in two clades: the clinical or pathogenic clade to refer to *S. brasiliensis*, *S. schenckii*, *S. globosa*, and *S. luriei* (former *S. schenckii* var. *luriei*) and the environmental clade, composed by *S. pallida* complex (*S. chilensis*, *S. mexicana*, *S. humicola*, and *S. pallida*) and the *S. stenoceras* complex (Rodrigues et al., 2020).

This fungal infection is globally distributed; however, actual incidence of the disease is difficult to measure, since sporotrichosis is not a notifiable disease in most countries (Barros et al., 2011; Gremião et al., 2021). The “classic” transmission of the etiological agent occurs through the skin by traumatic inoculation of the fungus present in vegetal or organic matter containing conidia of *Sporothrix* sp (Schubach et al., 2004).

On the other hand, the zoonotic transmission occurs through scratching, biting, or contact with exudates from cutaneous lesions of infected animals, mainly cats (Barros et al., 2011; Gremião et al., 2015; Pereira et al., 2015). These animals are the most affected by endemic that occurs in the city of Rio de Janeiro (Brazil) for more than 2 decades. They present respiratory signs, such as sneezing, which may be related to lesions located in the nasal region, including the mucous (Schubach et al., 2004). However, the most common lesions in cats are skin nodules and ulcers, and most of these lesions are located on the head and extremities of the limbs and tail (Rosser and Dunstan, 2006; Reis et al., 2016).

Several studies describe the global distribution of these species in cases of human and animal sporotrichosis in addition to environmental isolation (Feeney et al., 2007; Marimon et al., 2008; Madrid et al., 2009; Oliveira et al., 2011; Chakrabarti et al., 2015; Zhang et al., 2015; Mcguinness et al., 2016; New et al., 2019). Similarly, studies have been reported cases of sporotrichosis cases by species belonging to the environmental clade (Oliveira et al., 2011; Rodrigues et al., 2013a, 2016; Nesseler et al., 2019; Makri et al., 2020; Valeriano et al., 2020).

In Latin America, the south and southeast of Brazil have predominance of zoonotic transmission, through infected dogs and cats (Barros et al., 2011; Gremião et al., 2015). The increase in cases of sporotrichosis was neglected for several years, making a previously rare disease frequent and uncontrolled in Brazil. Socioeconomic inequalities, combined with the scarcity of health services, have facilitated the expansion of cases. In Rio de Janeiro, where the disease is considered a hyperendemic, there is an animal sporotrichosis control program that included free diagnosis and treatment; however, the control measures used were insufficient, in part due to underreporting of cases, as notification compulsory is carried out only by some municipalities (Gremião et al., 2020). On the other hand, the central-southern mountainous region of Peru (Abancay) is the second largest area of the disease, associated the classical transmission (Pappas et al., 2000; Ramírez Soto, 2015). Finally, the third area with the highest number of cases is the mountainous and central region of Mexico, where the infection occurs mainly by *S. schenckii* and in rare cases, the species *S. globosa*, mainly in women and children (Chakrabarti et al., 2015; Estrada-Castañón et al., 2018).

Despite the role of Brazil in this epidemic scenario is important to highlight that the second and third most endemic regions are China and South Africa, respectively, and beyond these regions, sporotrichosis occurs in Japan, Australia, India, the remaining Americas, and Europe, being less prevalent, except for the unique outbreak in France at the beginning of last century (Zhang et al., 2015).

In consequence of different factors that intervene in the diffusion and propagation of the disease, mode of distribution and evolution is essential to preserve these isolates, to guarantee strategies for controlling sporotrichosis. Therefore, culture collections represent centers for the conservation of *ex-situ* genetic resources whose main objectives are the preservation and supply of biological material and associated information for scientific and industrial research and development. According to the World Federation of Culture Collections, Brazil has more than 37,700 microbial cultures, stored in 53 different banks. Among the most important collections of fungal cultures in the country, the Collection of Cultures of Filamentous Fungi created in 1922, located at the Oswaldo Cruz Foundation, Rio de Janeiro, Brazil.

Based on this, the aim of this study was to analyze the global distribution of environmental isolates of *Sporothrix* spp., or causal agents of infection in humans and animals, identified by polyphasic taxonomy, and their percentage deposited in culture collections around the world, in the sense of better understand the distribution and diversity of these fungi.

## Methods

### Searching activities

Five bibliographic databases were searched (PubMed, Web of Science, Lilacs, Medline, and Scopus). The Search equation was described as follow: (((*Sporothrix brasiliensis*[Title/Abstract] OR *S. brasiliensis*[Title/Abstract] OR *Sporothrix schenckii*[Title/Abstract] OR *S. schenckii*[Title/Abstract] OR *Sporothrix globosa*[Title/Abstract] OR *S. globosa*[Title/Abstract] OR *Sporothrix mexicana*[Title/Abstract] OR *S. mexicana*[Title/Abstract] OR Sporothrix pallida[Title/Abstract] OR *S. pallida*[Title/Abstract] OR *Sporothrix albicans*[Title/Abstract] OR *S. albicans*[Title/Abstract] OR *Sporothrix luriei*[Title/Abstract] OR *S. luriei*[Title/Abstract] OR *Sporothrix complex*[Title/Abstract])) AND (PCR or Polymerase Chain Reaction or molecular OR calmodulin OR beta-Tubulin OR beta Tubulin OR Chitin Synthase[MeSH Terms])) AND (“2007/01/01”[Date - Publication]: “2023/06/01”[Date - Publication]).

### Screening process

According to PRISMA (Preferred Reporting Items for Systematic Reviews and Meta-Analyses) statement, accessed in http://www.prisma-statement.org, two independent reviewers screened titles and abstracts after excluding the repeated publications. The eligibility criteria followed to include the articles were (a) articles in English, (b) articles on animal sporotrichosis of human sporotrichosis and environmental isolates of *Sporothrix* sp. from 01-01-2007 to 12-31-2023, (c) all articles should identify the disease as sporotrichosis or reported the occurrence of isolation of *Sporothrix* sp. in environmental samples, and (d) it was necessary to identify the species; however, identification of the isolate’s location was not mandatory. The isolates described as unknown (not known) were analyzed and reported as unknown. The exclusion criteria used were the non-inclusion of the thesis, dissertations, monographs, publications with no strain identification (without a verification code), experimental model, and unavailable full texts.

The year 2006 was chosen to start the analysis, due to the description of new pathogenic species of *Sporothrix* sp., based on molecular and phenotypic studies that demonstrated intraspecific variability among the isolates morphologically identified as *S. schenckii*, indicating that this should not be considered a single species causing sporotrichosis, but rather a complex of species.

### Data extraction and epidemiological analysis

Two reviewers independently extracted the following variables: the number of the identified strain, country of origin, origin city (not obligatory), species identification, clinical or environmental clade, strain of origin, stored in collection, and specific collection (ATCC, CBS, CMW, IFM, IHEM, MUM, and IOC). These variables were chosen for the following reasons: geographic location, so that we could estimate the distribution of these species around the world. Identification of species by molecular methods, using the gold standard that is sequencing, in order to authenticate these species and correlate these data with their classification in the environmental or clinical clades, and their source of origin, since species belonging to the environmental clade, considered non-pathogenic or less virulent, have emerged as causal agents of sporotrichosis. Finally, the choice of collections was based on the official culture collections of the World Federation of Culture Collections. Data analysis was conducted in the R environment version 4.1.2.

## Results

### Study selection process

**Figure 1:**
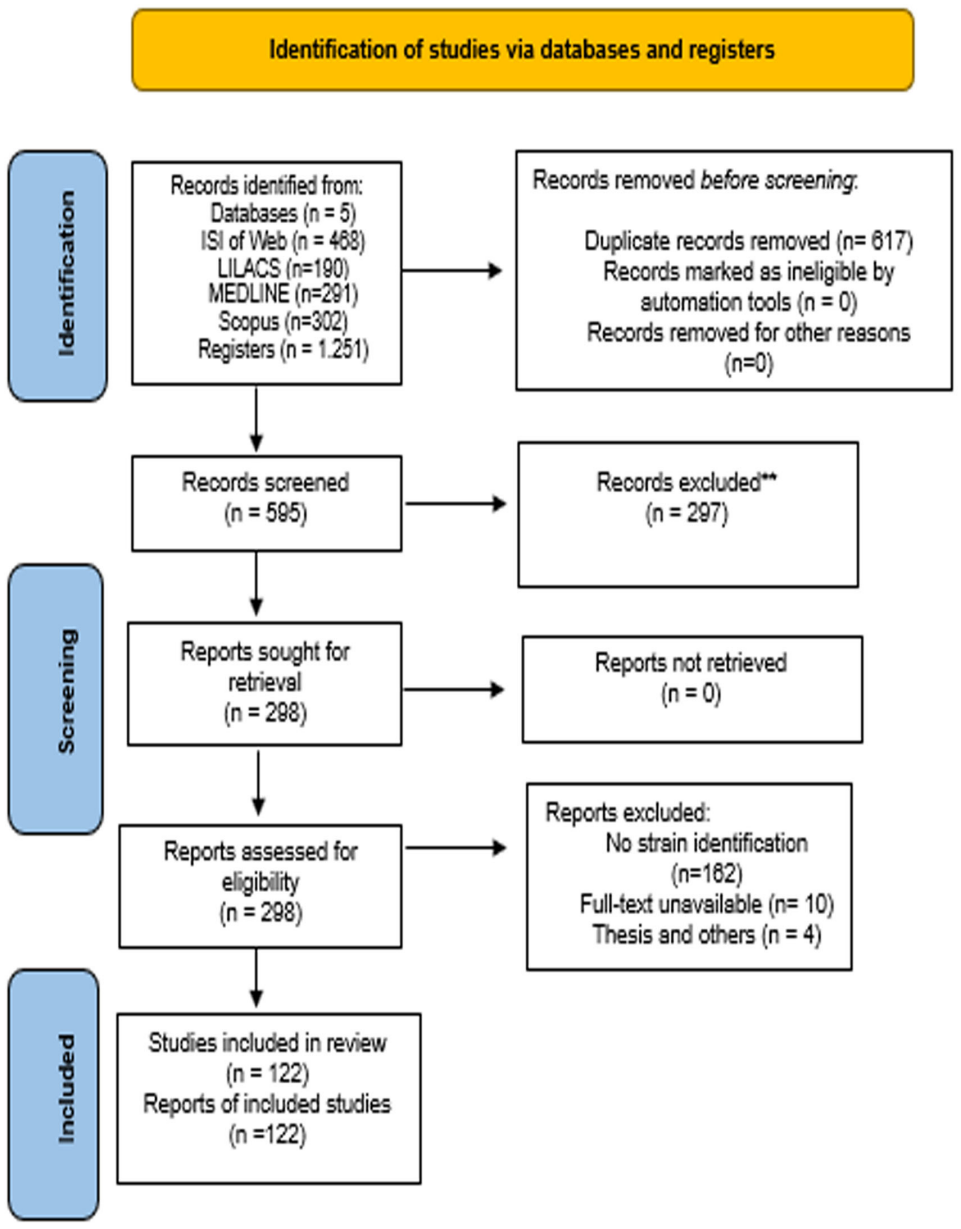
PRISMA 2020, flow diagram of the search and inclusion process in the study.

**Figure 1** shows the flowchart of the study selection process. Total of 122 articles were analyzed and, for the evaluation of these articles, were identified the number the strain, country of origin, city (not obligatory), species identification, clinical or environmental clade, source of the strain (human, animals, plants, soil, or unknown), as well as the number these strains in culture collections.

### Distribution of species by continent

**Figure 2:**
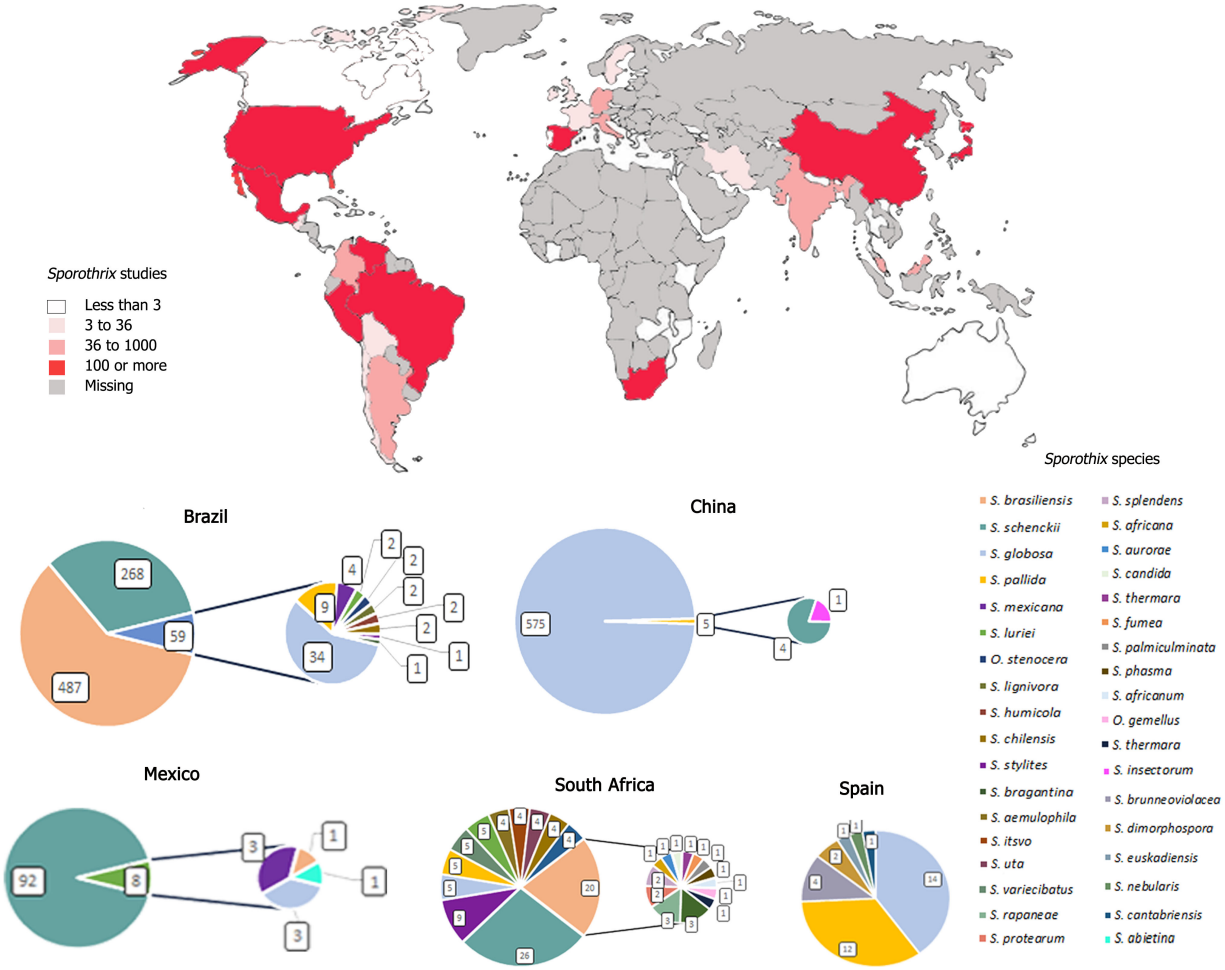
Number of *Sporothrix* sp. isolates described in the world from period of 2007–2023. The darkest green in the figure represents the largest number of isolates identified, in highest number in South America, followed by Asia, Africa, Europe, North America, Central America, and Oceania.

**Figure 2**; **Table 1** demonstrate the distribution of isolates of each by continent. South America was the continent where the highest number of isolates of sporotrichosis were reported, followed by Asia, North America, Africa, Europe, and Central America and Oceania were reported lower number of cases.

**Table 1:** Absolute number of the isolates in the countries.

Continent	Country	Species
North America (n= 148)	USA (n =47)	*S. brunneoviolacea* (n=2), *S. eucastanea* (n=1), *S. globosa* (n=7), *S. gossypina* (n=1), *S. rossii* (n=1), *S. schenckii* (n=35)
	Mexico (n= 100)	*S. abietina (n=1), S. brasiliensis (n= 1), S. globosa (n= 3), S. mexicana (n= 3), S. schenckii (n= 92)*
	Canada (n= 1)	*S. dimorphospora* (n= 1)
Central America (n= 4)	Costa Rica (n=1)	*S. schenckii* (n= 1)
	Guatemala (n= 3)	*S. globosa* (n= 1), *S. schenckii* (n=2)
South America (n= 1018)	Argentina (n=19)	*S. brasiliensis* (n=7), *S cabralli* (n=3*), S. epigloea (n= 1), S. lignivora* (n= 1), *S. schenckii* (n= 7)
	Bolivia (n=2)	*S. schenckii (n= 2)*
	Brazil (n=813)	*S. brasiliensis* (n= 487), *S. chilensis* (n=2), *S. globosa* (n= 34), *S. luriei* (n= 2), *S. mexicana* (n= 4), *S. pallida* (n= 9), *S. schenckii* (n= 268), *O. Stenocera* (n=2), *S. humicola* (n=2), *S. lignivora* (n=2), *S. stylites* (n=1)
	Chile (n= 12)	*S. chilensis* (n= 2), *S. dimorphospora* (n= 3), *S. dombeyi* (n= 1), *S. inflata* (n= 1), *S. pallida* (n= 1), *S. brasiliensis* (n= 4)
	Colombia (n=53)	*S. globosa* (n= 13), *S. schenckii* (n= 40)
	Peru (n=32)	*S. schenckii (n= 32)*
	Paraguay (n=2)	*S. brasiliensis* (n= 2*)*
	Venezuela (n= 85)	*S. globosa (n= 10), S. schenckii (n= 75)*
Europe (n= 103)	Austria (n=10)	*S. brunneoviolacea* (n= 2), *S. lunatum* (n= 1), *S. pallida* (n= 7)
	United Kingdom (n=8)	*S. globosa* (n= 1), *S. pallida (n= 2), S. humicola* (n=1), *S. brasiliensis* (n= 4)
	France (n=1)	*S. schenckii* (n= 1)
	Germany (n= 11)	*S. brunneoviolacea* (n= 1), *S. inflata* (n= 1), *S. globosa* (n= 1), *S. nivea*, (n= 1), *S. pallida* (n= 6), S. *schenckii* (n= 1)
	Greece (n=1)	*S. schenckii* (n= 1)
	Hungary (n=1)	*S. dentifunda* (n= 1)
	Italy (n=14)	*S. globosa* (n= 1), *S. mexicana* (n= 1), *S. pallida* (n= 7), *S. schenckii* (n= 5)
	Netherlands (=14)	*S. dimorphospora* (n= 2), *S. foliorum* (n= 1), *S. inflata* (n= 2), *S. humicola* (n= 1), *S. narcissi* (n= 1), *S. pallida* (n= 5), *S. schenckii* (n= 2)
	Norway (n=2)	*O. stenoceras* (n= 2)
	Poland (n=1)	*S. poliporicola* (n= 1)
	Portugal (n=2)	*S. globosa* (n= 1), *S. mexicana* (n= 1)
	Spain (n=35)	*S. brunneoviolacea* (n= 4), *S. cantabriensis* (n= 1), *S. dimorphospora* (n= 2), *S. euskadiensis* (n= 1), *S. globosa* (n= 14), *S. nebularis* (n= 1), *S. pallida* (n= 12)
	Sweeden (n=3)	*O. stenoceras* (n= 1), *S. inflata* (n= 1), *S. polyporicola* (n= 1)
Africa (n= 110)	Kenya (n= 1)	*O. stenoceras* (n= 1)
	Madagascar (n= 12)	*S. schenckii* (n= 12)
	Mozambique (n=1)	*S. schenckii* (n= 1)
	South Africa (n=94)	*S. aemulophila* (n= 4), *S. africana* (n= 1*), S. africanum* (n= 1), *S. aurorae* (n= 1*), S. candida* (n= 1), *S. fumea* (n= 1), *S. gemellus* (n= 1), *S. globosa* (n= 5), *S. humicola* (n= 3), *S. istvo* (n= 4), *S. lignivora* (n= 5), *S. luriei* (n= 4*), S. mexicana* (n= 4), *S. pallida* (n= 5), *S. palmiculminata* (n= 1)*, S. phasma* (n= 1*), S. protearum* (n= 2), *S. rapaneae* (n= 3), *S. schenckii* (n= 26), *S. splendens* (n= 2), *S. stylites* (n= 9), *S. thermara* (n= 1), *S. uta* (n= 4), *S. variecibatus* (n= 5)
	Zambia (n=2)	*S. protea-sedis* (n= 1)*, S. zambiensis* (n= 1)
Asia (n= 895)	Azerbaijan (n= 1)	*S. fusiforme* (n= 1)
	China (n=580)	*S. globosa (n= 575), S. insectorum* (n= 1), *S. schenckii (n= 4)*
	India (n=87)	*S. globosa* (*n= 87*)
	Iran (n=18)	*S. globosa* (n= 10), *S. schenckii* (n= 8)
	Korea (n= 8)	*S. globosa* (n= 8)
	Japan (n= 144)	*S. globosa* (n= 118), *S. nigrograna* (n= 1), *S. nivea* (n= 1), *S. pallida* (n= 2), *S. schenckii* (n= 22)
	Malasya (n=30)	*S. guttiliformis* (n= 1), *S. schenckii* (n= 29)
	Thailand (n= 27)	*S. schenckii* (n= 27)
Oceania (n= 4)	Australia (n=2)	*S. eucalyptigena* (n= 1), *S. pallida* (n=1)
	New Zealand (n=1)	*S. nothofagi* (n= 1)
	Tasmania (1)	*S. humicola* (n=1)

#### South America

A total of 1,019 isolates of *Sporothrix* sp. have been reported in seven countries in South America: Argentina, Brazil, Bolivia, Chile, Colombia, Paraguay, Peru, and Venezuela (**Table 1**). In the South American continent, the largest number of cases of sporotrichosis was identified in the study. Most of the strains identified in this evaluation were isolated from Brazil (814 strains), where the South and Southeast regions of the country are considered endemic areas of the disease.

The countries with the most reported cases in Latin America after Brazil were Venezuela with 85 isolates and Colombia with 53 isolates. Argentina (19 strains), Peru (32 strains), Chile (12 strains), Bolivia, and Paraguay (two strains) had the lowest number of cases. *S. brasiliensis* with 497 isolates was the specie with the highest number of identified isolates, followed by *S. schenckii* with 400 strains. The species of the environmental clade isolated on the mainland were *S. bragantina*, *S. cabrallii*, *S. chilensis*, *S. dimorphospora*, *S. dombeyi*, *S. epigloea*, *S. humicola*, *S. inflata*, *S. lignivora*, *S. mexicana*, *S. pallida*, and *S. stylites*. The species were isolated from humans, animals (cat and dog), and environment (plant and soil). One hundred twenty-nine isolates from the South American continent are stored in a collection (13%). The *CAL* gene (58%) was the most usable molecular method, followed by T3B *fingerprinting* (29%), *ITS* region (15%), β-tubulin (6%), *CHS* gene (3%), and other molecular methods (23%).

#### North America

North American countries reported a total of 148 cases in the period evaluated (**Table 1**). The country with the most *Sporothrix* sp. isolates was Mexico (100 strains identified), followed by the United States (47 isolates). In Canada, one isolate was identified. Ninety-three percent of the isolates were from clinical source. *S. schenckii* (127 strains) was the most isolated in this continent. Seven percent of the species identified on the North American continent belong to the environmental clade: *S. brunneoviolacea*, *S. dimorphospora*, *S. eucastanea*, *S. gossypina*, *S. abietina*, *S. mexicana*, and *S. rossii.* These species were isolated human, animals, and environmental. North American 19 isolates are stored in a collection (13%). For the identification of the species, most used molecular method was *CAL* gene (68%), followed by β-tubulin (11%), *ITS* region (9%), *CHS* gene (3%), and others molecular methods (10%).

#### Central America

Isolation of four *Sporothrix* sp. strains was reported from Central America (**Table 1**): Guatemala and Costa Rica, three isolates and one isolate were described, respectively. A hundred percent of the isolates were from the clinical clade, and *S. schenckii* was the species with the highest number of isolates (three strains). One strain of *S. globosa* was isolated and all the samples were collected from human cases. None of the isolates identified on this continent are stored in a collection. The *CAL* gene (75%) was the most usable molecular method, followed by, *ITS* region and β-tubulin (25%).

#### Asia

A total of 895 isolates were found of Asian continent, from eight Asian countries: Azerbaijan, China, India, Iran, Korea, Malaysia, Japan, and Thailand (**Table 1**). China was the country in which were reported the most cases (580 isolates), followed by Japan (144 isolates). The most isolated species on the Asian continent were *S. globosa* (803 isolates) and *S. schenckii* (94 isolates). Nighty-nine percent of the isolates were from clinical source. From the environmental clade were collected 1% of species on the continent (*S. fusiforme*, *S. insectorum*, *S. guttiliformis*, *S. nigrograna*, *S. nivea*, *S. guttiliformis*, and *S. pallida)*, isolated from plant, soil, and cats. In this continent, 99 isolates are stored in a collection (11%). For the identification of the species, the most used molecular method was *CAL* gene with 68%, followed by *ITS* region (38%), β-tubulin (17%), *Chitin synthase* gene (*CHS)* (1%), and other molecular methods (9%).

#### Europe

The total strains of *Sporothrix* sp. reported in Europe were 103 isolates (**Table 1**). The country with the highest number of isolates was Spain (35 strains), followed by Italy and Netherlands (14 strains), Germany (11 strains), and Austria (10 strains). In this continent was observed the identification of higher percentages of species from the environmental than clinical clade, with 71% and 29%, respectively. In the environmental clade, the genera *Ophiostoma stenoceras* and *S. brunneoviolacea*, *S. cantabriensis*, *S. dentifunda*, *S. dimorphospora, S. euskadiensis*, *S. foliorum*, *S. humicola*, *S. inflata*, *S. luneta*, *S. lunatum*, *S. mexicana*, *S. narcissi*, *S. nebularis*, *S. nivea*, *S. pallida*, *S. polyporicola, and S. prolifera* were identified. Samples were collected from humans, animals, and environment. On the European continent, 49 isolates are stored in a collection (49% of the isolates). For the identification of the species, most used molecular method was *CAL* gene (73%), followed by *ITS* region (40%), β-tubulin (35%), *CHS* gene (31%), T3B fingerprinting (1%), and other molecular methods (22%).

#### Africa

Isolation of 111 *Sporothrix* sp. strains were reported in five countries of the African continent: Mozambique and Kenya (one isolate), Zambia (two isolates), Madagascar (12 isolates), and South Africa, the country with the highest number of cases (95 isolates) (**Table 1**). The species with the highest number of isolates found on the African continent was *S. schenckii* (39 isolates). Sixty-eight percent of species were environmental clade, *S. aemulophila*, *S. africana*, *S. africanum*, *S. aurorae*, *S. candida*, *S. curviconia*, *S. fumea*, *S. gemella*, *S. gemellus*, *S. humicola*, *S. itsvo*, *S. lignivora*, *S. mexicana*, *S. pallida*, *S. palmiculminata*, *S. phasma*, *S. protea-sedis*, *S. protearum*, *S. rapaneae*, *S. splendens*, *S. stylites*, *S. thermara*, *S. uta*, *S. variecibatus*, *S. zambiensis*, and one isolate of the genus *Ophiostoma stenoceras.* The samples were collected from human, animals, and environmental. On the African continent, 80 isolates are stored in a collection; therefore, 73% of isolates on the continent were preserved. The identification at the species level by the *ITS* region was 62%, followed by β-tubulin (58%), *CAL* gene (41%), and other molecular methods (16%).

#### Oceania

In Australia, Tasmania and New Zealand were reported the isolation of four different *Sporothrix* sp. strains, belonging to the environmental clade (**Table 1**). *S. eucalyptigena* and S. pallida were isolated from Australia, *S. humicola* from Tasmania and *S. nothofagi* from New Zealand. Strains were isolated from the plant and animals. Fifty percent the isolates were the stored in a collection. For the identification of the species, molecular methods were used: Sequencing of specific regions of the rRNA *ITS*, β-tubulin (*Beta*), *Calmodulin* gene (*CAL*), and others molecular methods with 100%.

## Discussion

The present study shows the isolation reports of 2.284 *Sporothrix* sp. strains in the world for the 2007–2023 period. Most of the isolates were reported from South America (*n* = 938/44%), followed by Asia (*n* = 865/40%), and Oceania (*n* = 4/0.18%) with less frequency. After the description of new species from the genus *Sporothrix* sp., the identification of clinical isolates has been performed worldwide, especially in regions where many sporotrichosis cases occur (Boechat et al., 2022), for example, in southeastern Brazil, an area of zoonotic epidemic of sporotrichosis (Gremião et al., 2017) and Japan (Chakrabarti et al., 2015). Corroborating with our data, where countries with number of isolates were Brazil (*n* = 814), China (*n* = 580), Japan (*n* = 144), and South Africa (*n* = 95). On the other hand, data from many countries of Africa and Europe are unavailable.

Culture Collections, upon receiving an isolate, begin the process of maintaining its characteristics, with the purpose of maintaining the viability, morphological, physiological, and genetic characteristics of the isolates for long periods, allowing the use of these isolates in important themes in the field of mycology. Depending on the species being preserved and the storage method fungal isolates are preserved in culture collections and the storage time varies around 10 years or more (Nakasone et al., 2004; Rabello et al., 2022). According with study, 378 strains were stored in collection, the continents that most preserved strains were, Africa (*n* = 80/73%), followed by Europe (*n* = 49/63%), Oceania (*n* = 2/50%), Asia (*n* = 99/11%), South America (*n* = 129/14%) and North America (*n* = 19/13%). It is important to reinforce that our study evaluated only isolates stored in culture collections, whose data are published in articles, and were not considered “internal” or “non-public” repositories due to lack of knowledge about these data.

The culture collections that most stored *Sporothrix* sp. strains were CBS Filamentous fungi and Yeast Collection (*n* = 197) and CMW—Culture Collection of Innovation Africa from the University of Pretoria (*n* = 71), respectively. In the CBS collection, the continents that deposited it most isolated were South America and Asia, although this collection is European. Moreover, in the CMW collection, which is in South Africa, it corroborates our data that the African continent has preserved the largest number of strains in this collection. In relation to the other continents of the world, the number of strains stored in the two collections was proportional.

It should be noted that, although several authors report cases of sporotrichosis worldwide (Chakrabarti et al., 2015; Rodrigues et al., 2020), there are insufficient numerous of strains preserved in collections. We also highlight that Brazil, followed by China, are the two countries with the highest number of described isolates; however, these countries do not contain the highest percentages of deposits in biological collections. In our study, we identified that 83% of *Sporothrix* sp. are not stored in a culture collection and only 17% are preserved for possible future studies.

Phylogenetic analysis of *Sporothrix* species has traditionally been carried out using sequencing data of single or multiple conserved genes, most notably *CHS*, β-tubulin, and the *CAL* gene; the latter is considered the reference standard for molecular identification of species of the genus *Sporothrix* sp (Marimon et al., 2007; New et al., 2019). The molecular method most used in Europe, Asia, North, Central, and South America was the identification of species by the *CAL* gene, and African continent was the identification by sequencing of specific regions of the rRNA ITS. This study used only articles that performed molecular characterization to identify species of the genus *Sporothrix* sp.

It is necessary to emphasize that, although several authors report the existence of cases of sporotrichosis throughout the world (Chakrabarti et al., 2015; Rodrigues et al., 2020), there is not enough data on the molecular epidemiology of the species. Mainly on animal sporotrichosis and isolation of *Sporothrix* sp. In the environmental samples in which our study identified insufficient data on the molecular identification, constituting one of the limitations of our study. Therefore, further studies about animal and human sporotrichosis, the presence and isolation of this fungus in the environment and molecular identification of species are necessary. Even though mycoses are neglected diseases, pandemics such as COVID-19 reinforce the need for more epidemiological studies using molecular tools, since the definition, identification, and monitoring of species potentially pathogenic to humans and animals is advocated by the concept “One health,” promotes the integration between public policies and human health, animal, and environment.

Although the absolute number of isolates deposited in collections is higher in South America, we must consider that the African continent has the highest percentages of isolates in collections. Furthermore, contrary to what was observed in South America, where most of the isolates are of clinical origin, especially due to the hyperendemic sporotrichosis in Rio de Janeiro, Brazil, on the African continent were observed a predominance of isolates of environmental origin (66 isolates), the which reinforces the relative risk offered by these species.

Sporotrichosis on the African continent showed outbreaks in the 40s (Quintal, 2000) and an outbreak in a gold mine in South Africa (Govender et al., 2015); however, there is no evidence of the disease’s reemergence on the continent, which may justify the lower number of clinical cases in the region. In comparison, South America had a higher prevalence of strains of human origin (673 strains) and animals (177 strains). Although South American countries are underdeveloped countries, there is a greater investment in the identification and confirmation of the disease, mainly in the identification of species through molecular methods. According to Albuquerque et al. (2020), Brazil is the current leader in annual publications on sporotrichosis in the world.

In our study, the most isolated species in the world was *S. globosa* (889 isolates), followed by *S. schenckii* (684 isolates), and *S. brasiliensis* (505 isolates). According to Zhang and collaborators (Zhang et al., 2015), most frequent species in the world are *S. globosa* and *S. schenckii* since *S. brasiliensis* species is abundant only in South America.

According to Alzuguir et al. (2019), social and geoecological factors can affect the incidence and prevalence of fungal diseases. For example, the increase in temperature induces greater microbial adaptation, since its geographic expansion promotes an increase in contact with the host. The effects of climate change create cycles of dispersal of fungal pathogens by nature, due to the increase in evaporation, clouds, and precipitation, which lead to extreme winds, potential dispersers of the fungi (Mendes et al., 2017). In addition, human activity can also contribute to the emergence of fungal diseases. In this context, according to epidemiological studies, was identified that *S. brasiliensis* is dependent on feline hosts for its emergence in southern and southeastern Brazil, as it is seen that the increase in the number of cases in cats is usually followed by an increase in the number of human cases (Rodrigues et al., 2013b; Rodrigues et al., 2014). Thus, One Health is the key to effective surveillance and successful disease control. Coordinated actions between veterinarians, laboratory professionals, surveillance authorities, and other health professionals will ensure broader investigations and promote prevention, detection, and assistance for human and animal cases (Gremião et al., 2020).

Finally, we would like to highlight the huge discrepancy regarding fungal species. It is estimated that there are over 1.5 million species of fungi in the world, but only about 70,000 have ever been formally described (Hawksworth, 2001; Casadevall, 2005), only 17% of the strains of *Sporothrix* sp. isolated in the world are preserved in a culture collection. More than 83% of the isolates described in this study are not stored in a biological collection, which can lead to contamination and loss of these isolates. Thus, we emphasize the importance of depositing in collections, as they are repositories of information on biodiversity that have facilities and expertise to ensure the correct identification of species, and to minimize the genetic drift that often occurs because of the repetitive transfer of strains, maintaining, thus, pure and viable cultures to be used in the future for experimental, educational, industrial, or comparative studies.

## Conclusion

Our study confirmed the difficulty in obtaining the frequency of Sporothrix species stored in culture collections around the world. However, one of the limitations of the study was the lack of data on the molecular identification of animal and human sporotrichosis and isolation of *Sporothrix* sp. in environmental samples.

Furthermore, we observed that the deposit in collections is not proportional to the isolation of fungal species, as is the case in countries with a greater number of isolates, such as Brazil and China (with 814 and 580 isolates, respectively), which are not the countries with the highest number of isolates. deposit percentages. This systematic review analyzed the geographic distribution of species causing sporotrichosis identified by polyphasic taxonomy. These data reinforce the need to use these tools to identify and monitor potential pathogens of different origins, to strengthen the “One Health Concept,” which acts as a promoter of health policies based on the integration between men, animals, and the environment. We also consider the importance of the environment as part of this whole, since climate change can be considered determining factors in the frequency and distribution of these species. However, future studies will be needed to understand the impact of climate change on the global epidemiology of sporotrichosis. We highlight the importance of preserving organisms in culture collections to guarantee their survival, stability, and purity for prolonged periods of time, conserving genetic characteristics and physiological/morphological properties, mainly for use in future studies of these isolates.

## Author contributions

DM: Writing – review & editing, Writing – original draft, Methodology. RC: Writing – review & editing, Software, Methodology. MR-A: Writing – review & editing, Methodology, Investigation, Formal Analysis. DC-M: Writing – review & editing, Writing – original draft, Validation, Formal Analysis, Data curation. JC: Writing – review & editing, Methodology, Formal Analysis, Data curation. RM: Writing – review & editing, Validation. MO: Writing – review & editing, Supervision, Resources, Project administration, Funding acquisition.
