# Supplementary material for: Systematic review of literature to evaluate global distribution of species of the Sporothrix genus stored in culture collections
Source: Front Cell Infect Microbiol. 2024 May 17;14:1382508. doi: 10.3389/fcimb.2024.1382508 (PMC11140055; doi:10.3389/fcimb.2024.1382508)
Supplement: Supplementary file 1 [file Table_1.docx]

Supplementary Table 1: Summary of studies included in the present systematic review.

| **Year of publication** | **Authors** | **Title** | **Country of the study*** | **Molecular Methods used** |
| --- | --- | --- | --- | --- |
| 2007 | Marimon *et al.* | *Sporothrix brasiliensis, S-globosa*, and *S. mexicana*, three new *Sporothrix* species of clinical interest. | Spain | *Cal* |
| 2008 | De Meyer *et al.* | Taxonomy and phylogeny of new wood- and soil inhabiting *Sporothrix* species in the *Ophiostoma stenoceras-Sporothrix schenckii* complex. | South Africa | *Beta; ITS* |
| 2008 | Marimon *et al.* | *Sporothrix luriei*: a rare fungus from clinical origin. | Spain | *Beta; Cal; CHS* |
| 2009 | Madrid *et al.* | *Sporothrix globosa*, a pathogenic fungus with widespread geographical distribution. | Spain | *Cal* |
| 2010 | Ramírez et al. | Esporotricosis cutánea fija en un adolescente causada por Sporothrix schenckii (sensu stricto) y revisión comparativa de la bibliografía*.* | Mexico | *Cal* |
| 2010 | Oliveira *et al.* | Sporotrichosis Caused By *Sporothrix* *globosa* in Rio De Janeiro, Brazil: Case Report. | Brazil | *Cal* |
| 2010 | Madrid *et al.* | *Sporothrix brunneoviolacea* and *Sporothrix dimorphospora*, two new members of the Ophiostoma stenoceras-*Sporothrix schenckii* complex. | Spain | *Beta; ITS* |
| 2011 | Arechavala *et al.* | Esporotricosis diseminada con compromiso cutáneo y visceral. | Argentina | *Cal* |
| 2011 | Dias *et al.* | Sporotrichosis Caused by *Sporothrix* *mexicana*, Portugal. | Portugal | *Cal* |
| 2011 | Oliveira *et al.* | Phenotypic and Molecular Identification of *Sporothrix* Isolates from an Epidemic Area of Sporotrichosis in Brazil. | Brazil | *Cal* |
| 2011 | Oliveira *et al.* | Antifungal Susceptibilities of *Sporothrix* *albicans*, *S. brasiliensis*, and *S. luriei* of the *S. schenckii* Complex Identified in Brazil. | Brazil | *Cal* |
| 2011 | Romeo *et al.* | New Insight into Molecular Phylogeny and Epidemiology of *Sporothrix* *schenckii* Species Complex Based on Calmodulin-Encoding Gene Analysis of Italian Isolates. | Italy | *Cal; CHS* |
| 2011 | Zhang *et al.* | Variation in Genotype and Higher Virulence of a Strain of *Sporothrix* *schenckii* Causing Disseminated Cutaneous Sporotrichosis. | China | *Beta; CHS; ITS; Other* |
| 2012 | Oliveira *et al.* | Rapid Identification of *Sporothrix* Species by T3B Fingerprinting. | Brazil | *Cal; T3B* |
| 2012 | Silva-Vergara *et al.* | Case Report: Disseminated *Sporothrix* *brasiliensis* Infection with Endocardial and Ocular Involvement in an HIV-Infected Patient. | Brazil | *Cal* |
| 2013 | Castro *et al.* | Differences in Cell Morphometry, Cell Wall Topography and Gp70 Expression Correlate with the Virulence of *Sporothrix* *brasiliensis* Clinical Isolates | Brazil | *Cal* |
| 2013 | Oliveira *et al.* | Molecular identification of *Sporothrix* species involved in the first familial outbreak of sporotrichosis in the state of Espírito Santo, southeastern Brazil. | Brazil | *Cal* |
| 2013 | Fernandes *et al.* | Characterization of virulence profile, protein secretion and immunogenicity of different *Sporothrix schenckii* sensu stricto isolates compared with *S. globosa* and *S. brasiliensis* species. | Brazil | *Cal; ITS* |
| 2013 | Kano *et al.* | *Sporothrix schenckii* (sensu strict *S. globosa*) mating type 1-2 (MAT1-2) gene. | Japan | *Cal* |
| 2013 | Orofino-Costa *et al.* | Pulmonary cavitation and skin lesions mimicking tuberculosis in a HIV negative patient caused by *Sporothrix brasiliensis*. | Brazil | *Cal* |
| 2013 | Rodrigues *et al.* | Emergence of pathogenicity in the *Sporothrix schenckii* complex. | Brazil | *Cal* |
| 2013 | Rodrigues *et al.* | Phylogenetic Analysis Reveals a High Prevalence of *Sporothrix* *brasiliensis* in Feline Sporotrichosis Outbreaks. | Brazil | *Cal; Other* |
| 2013 | Yu *et al.* | Phenotypic and molecular identification of *Sporothrix* isolates of clinical origin in Northeast China | China | *Cal* |
| 2014 | Almeida-Paes *et al.* | Sporotrichosis in Rio de Janeiro, Brazil: *Sporothrix brasiliensis* Is Associated with Atypical Clinical Presentations | Brazil | *Cal* |
| 2014 | Choappa *et al.* | Aislamiento de *Sporothrix pallida* complex en muestras clínicas y ambientales de Chile. | Chile | *Beta* |
| 2014 | Oliveira *et al.* | First autochthone case of sporotrichosis by *Sporothrix globosa* in Portugal. | Brazil | *Beta; Cal; T3B* |
| 2014 | Liu *et al.* | Molecular identification of *Sporothrix* clinical isolates in China | China | *Beta; ITS* |
| 2014 | Stopiglia *et al.* | Antifungal susceptibilities and identification of species of the *Sporothrix* *schenckii* complex isolated in Brazil. | Brazil | *Cal* |
| 2014 | Rodrigues *et al.* | *Sporothrix schenckii* sensu stricto Isolated from Soil in an Armadillo's Burrow. | Brazil | *Cal* |
| 2014 | Rodrigues *et al.* | Genotyping species of the *Sporothrix schenckii* complex by PCR-RFLP of calmodulin. | Brazil | *Cal; RFLP Cal* |
| 2014 | Rodrigues *et al.* | Genetic diversity and anti fungal susceptibility profiles in causative agents of sporotrichosis. | Brazil | *Cal; ITS* |
| 2014 | Rodrigues *et al.* | Emerging sporotrichosis is driven by clonal and recombinant *Sporothrix* species. | Brazil | *Cal; ITS; Other* |
| 2014 | Sasak *et al.* | Chromosomal Polymorphism in the *Sporothrix schenckii* Complex. | Brazil | *Beta; Cal; CHS; ITS; Other* |
| 2014 | Teixeira *et al.* | Comparative genomics of the major fungal agents of human and animal Sporotrichosis: *Sporothrix schenckii* and *Sporothrix brasiliensis*. | Brazil | *Other* |
| 2014 | Zhou *et al. et al.* | Global ITS diversity in the *Sporothrix schenckii* complex | China | *Beta; ITS* |
| 2015 | Macedo *et al.* | Dacryocystitis due to *Sporothrix brasiliensis*: a case report of a successful clinical and serological outcome with low-dose potassium iodide treatment and oculoplastic surgery. | Brazil | *Cal* |
| 2015 | Kano *et al.* | The MAT1-1:MAT1-2 Ratio of *Sporothrix globosa* Isolates in Japan. | Japan | *Cal; ITS; Other* |
| 2015 | Oliveira *et al.* | Development and optimization of a new MALDI-TOF protocol for identification of the *Sporothrix* species complex. | Brazil | *Cal; Other* |
| 2015 | Borba-Santos *et al.* | Susceptibility of *Sporothrix* *brasiliensis* isolates to amphotericin B, azoles, and terbinafine | Brazil | *Cal* |
| 2015 | Camacho *et al.* | Molecular epidemiology of human sporotrichosis in Venezuela reveals high frequency of *Sporothrix globosa* | Venezuela | *Cal; ITS* |
| 2015 | Araujo *et al.* | Human sporotrichosis beyond the epidemic front reveals classical transmission types in Espírito Santo, Brazil | Brazil | *ITS* |
| 2015 | Freitas *et al.* | Increase in virulence of *Sporothrix brasiliensis* over five years in a patient with chronic disseminated sporotrichosis | Brazil | *Beta; CHS; T3B* |
| 2015 | Govender *et al.* | An Outbreak of Lymphocutaneous Sporotrichosis among Mine-Workers in South Africa | South Africa | *Cal* |
| 2015 | Hu *et al.* | Construction and analysis of the cDNA subtraction library of yeast and mycelial phases of *Sporothrix globosa* isolated in China: identification of differentially expressed genes | China | *ITS* |
| 2015 | Kano *et al.* | Molecular typing of *Sporothrix schenckii* isolates from cats in Malaysia | Malaysia | *Cal; ITS; Other* |
| 2015 | Musvuugwa *et al.* | New species of Ophiostomatales from Scolytinae and Platypodinae beetles in the Cape Floristic Region, including the discovery of the sexual state of Raffaelea | South Africa | *Beta; Cal; ITS* |
| 2015 | Oliveira *et al.* | Evaluation of T3B fingerprinting for identification of clinical and environmental *Sporothrix* species | Brazil | *Cal; T3B* |
| 2015 | Rodrigues *et al.* | Rapid Identification of Emerging Human-Pathogenic *Sporothrix* Species with Rolling Circle Amplification | Brazil | *Cal; ITS; Other* |
| 2015 | Xirotagaros *et al.* | Imported lymphocutaneous sporotrichosis in Greece | Greece | *Cal; Other* |
| 2015 | Zhang *et al.* | Phylogeography and evolutionary patterns in *Sporothrix* spanning more than 14 000 human and animal case reports | China | *Cal; ITS; Other* |
| 2016 | Almeida-Paes *et al.* | Refractory sporotrichosis due to *Sporothrix brasiliensis* in humans appears to be unrelated to in vivo resistance | Brazil | *T3B* |
| 2016 | De Beer *et al.* | The divorce of *Sporothrix* and Ophiostoma: solution to a problematic relationship | South Africa | *Beta; Cal; ITS; Other* |
| 2016 | Errasti *et al.* | Three new species of Ophiostomatales from Nothofagus in Patagonia | Argentina | *Beta; ITS; Other* |
| 2016 | Gompertz *et al.* | Atypical Clinical Presentation of Sporotrichosis Caused by Sporothrix globosa Resistant to Itraconazole | Brazil | *Cal* |
| 2016 | Mahmoudi *et al.* | *Sporothrix schenckii* complex in Iran: Molecular identification and antifungal susceptibility | Iran | *Cal* |
| 2016 | Musvuugwa *et al.* | Wounds on Rapanea melanophloeos provide habitat for a large diversity of Ophiostomatales including four new species | South Africa | *Beta; Cal; ITS* |
| 2016 | Rodrigues *et al.* | *Sporothrix chilensis* sp. nov. (Ascomycota: Ophiostomatales), a soil-borne agent of human sporotrichosis with mild-pathogenic potential to mammals | Brazil | *Beta; Cal; ITS; Other* |
| 2016 | Suzuki *et al.* | Studies in Phylogeny, Development of Rapid Identification Methods, Antifungal Susceptibility, and Growth Rates of Clinical Strains of *Sporothrix schenckii* Complex in Japan | Japan | *Cal* |
| 2016 | Zhang *et al.* | Two cases of sporotrichosis of the right upper extremity in right-handed patients with diabetes mellitus | China | *Cal* |
| 2017 | Nath *et al.* | Molecular Identification and Phenotypic Characterisation of Sporothrix globosa from Clinical Cases of Eastern Assam, North-east India | India | *Cal* |
| 2017 | Zhao *et al.* | Genetic variation of Sporothrix globosa isolates from diverse geographic and clinical origins in China | China | *Cal* |
| 2017 | Vetorrato *et al.* | Sporotrichosis by Sporothrix schenckii senso stricto with itraconazole resistance and terbinafine sensitivity observed in vitro and in vivo: Case report | Brazil | *Cal* |
| 2017 | Galati *et al.* | Sporotrichosis­ cat as the source of familiar infection outbreak in Guarulhos, São Paulo: a case report/ Esporotricose ­ gato como fonte de infecção em foco epidêmico familiar em Guarulhos, São Paulo: relato de caso | Brazil | *Cal* |
| 2018 | Cordoba *et al.* | Molecular identification and susceptibility profile of Sporothrix schenckii sensu lato isolated in Argentina | Argentina | *Cal* |
| 2018 | Boechat *et al.* | Feline sporotrichosis: associations between clinical-epidemiological profiles and phenotypic-genotypic characteristics of the etiological agents in the Rio de Janeiro epizootic area | Brazil | *T3B* |
| 2018 | Fernandes *et al.* | A case of disseminated sporotrichosis caused by *Sporothrix brasiliensis* | Brazil | *ITS* |
| 2018 | Gomez *et al.* | Draft Genome Sequences of Two *Sporothrix schenckii* Clinical Isolates Associated with Human Sporotrichosis in Colombia | Colombia | *Cal* |
| 2018 | Fichman *et al.* | Cryosurgery for the treatment of cutaneous sporotrichosis in four pregnant women. | Brazil | *T3B* |
| 2018 | Takazawa *et al.* | Case of pyoderma gangrenosum-like sporotrichosis caused by *Sporothrix globosa* in a patient with ulcerative colitis | Japan | *ITS, Cal* |
| 2018 | Boechat *et al.* | Feline sporotrichosis: associations between clinical-epidemiological profiles and phenotypic-genotypic characteristics of the etiological agents in the Rio de Janeiro epizootic area | Brazil | *T3B* |
| 2018 | Rojas *et al.* | Molecular Identification, Antifungal Susceptibility, and Geographic Origin of Clinical Strains of *Sporothrix schenckii* Complex in Mexico | Mexico | *Cal* |
| 2018 | Ochoa-Reyes *et al.* | Esporotricosis del pabellón auricular: comunicación de un caso atípico simulando una celulitis bacteriana/ Auricular sporotrichosis: atypical case report simulating bacterial cellulitis | Mexico | *Cal* |
| 2019 | Azam *et al.* | Molecular epidemiology of *Sporothrix schenkii* isolates in Malaysia | Malasya | *Cal* |
| 2019 | Nesseler *et al.* | *Sporothrix humicola (*Ascomycota*: Ophiostomatales*) – A soil-borne fungus with pathogenic potential in the eastern quoll (Dasyurus viverrinus) | Germany | *ITS, Beta, Cal* |
| 2019 | Oliveira *et al.* | A case of sporotrichosis caused by different *Sporothrix brasiliensis* strains: mycological, molecular, and virulence analyses | Brazil | *Cal, Beta, CHS* |
| 2019 | Wang *et al.* | A Patient With Sporotrichosis Diagnosed By Molecular Biology Combined With Traditional Methods | China | *Cal* |
| 2019 | Thomson *et al.* | An atypical cause of sporotrichosis in a cat | Australia | *ITS, Beta* |
| 2019 | Flórez-Muñoz *et al*. | Molecular Identification and Antifungal Susceptibility of Clinical Isolates of *Sporothrix schenckii* Complex in Medellin, Colombia | Colombia | *Cal* |
| 2019 | Eyer-Silva *et al.* | Palate ulcer, uvular destruction and nasal septal perforation caused by *Sporothrix brasiliensis* in an HIV-infected patient | Madagascar | *Cal* |
| 2019 | Rosaemolida *et al.* | Sporotrichosis in the Highlands of Madagascar, 2013-2017. | Madagascar | *ITS* |
| 2019 | Matos *et al.* | Identification by MALDI-TOF MS of *Sporothrix brasiliensis* Isolated from a Subconjunctival Infiltrative Lesion in an Immunocompetent Patient | Brazil | *MALDI-TOF* |
| 2019 | Kim *et al.* | Molecular Identification of Human Sporotrichosis in Korea | Korea | *ITS* |
| 2019 | Gong *et al.* | Population Structure and Genetic Diversity of *Sporothrix globosa* in China According to 10 Novel Microsatellite Loci. | China | *ITS* |
| 2020 | Spanamberg *et al.* | *Sporothrix brasiliensis* on cats with skin ulcers in Southern Brazil | Brazil | *Cal* |
| 2020 | Boechat *et al.* | Canine sporotrichosis: polyphasic taxonomy and antifungal susceptibility profiles of *Sporothrix* species in an endemic area in Brazil | Brazil | *T3B* |
| 2020 | Macêdo-Sales *et al.* | Coinfection of domestic felines by distinct *Sporothrix brasiliensis* in the Brazilian sporotrichosis hyperendemic area | Brazil | *Cal, Beta* |
| 2020 | Makri *et al.* | First case report of cutaneous sporotrichosis (*Sporothrix* species) in a cat in the UK | United Kingdom | *Cal, Beta* |
| 2020 | Fichman *et al.* | Zoonotic sporotrichosis in renal transplant recipients from Rio de Janeiro, Brazil | Brazil | *Cal* |
| 2020 | Reinprayoon *et al.* | Conjunctival sporotrichosis from cat to human: Case report | Brazil | *Cal* |
| 2020 | Alves *et al.* | Fatal pulmonary sporotrichosis caused by *Sporothrix brasiliensis* in Northeast Brazil | Brazil | *Beta, Cal* |
| 2020 | Valeriano *et al.* | Is *Sporothrix chilensis* circulating outside Chile? | Brazil | *Beta* |
| 2020 | Ramírez-Soto *et al.* | Molecular identification of *Sporothrix* species in a hyperendemic area in Peru | Peru | *Cal* |
| 2020 | Bonifaz *et al.* | Atypical sporotrichosis related to *Sporothrix* *mexicana* | Mexico | *Cal* |
| 2020 | Gu *et al.* | Fixed cutaneous sporotrichosis in a patient with numerous fungal elements | China | *Cal* |
| 2020 | Caballero *et al.* | Value of molecular techniques and risk factors in the diagnosis and evolution of sporotrichosis. About 2 cases of *Sporothrix brasiliensis* and *S. globosa* | Brazil | *Cal* |
| 2021 | Filho *et al.* | A novel *Sporothrix brasiliensis* genomic variant in Midwestern Brazil: evidence for an older and wider sporotrichosis epidemic | Brazil | *Cal* |
| 2021 | Araujo *et al.* | Case Report: Invasive Sinusitis due to *Sporothrix brasiliensis* in a Renal Transplant Recipient | Brazil | *Cal* |
| 2021 | Barreto *et al.* | Geographical distribution and ecological niche modeling of the etiological agents of human sporotrichosis in Venezuela | Brazil | *Cal* |
| 2021 | Liu *et al.* | Fixed Cutaneous Sporotrichosis Due to *Sporothrix globosa* | China | *ITS, Beta* |
| 2021 | Rudramurthy *et al.* | Phenotypic and molecular characterisation of *Sporothrix globosa* of diverse origin from India | India | *Cal* |
| 2021 | Sendrasoa *et al.* | Osteoarticular sporotrichosis in an immunocompetent patient | Madagascar | *Cal* |
| 2021 | Martiniz-Herrea *et al.* | Uncommon Clinical Presentations of Sporotrichosis: A Two-Case Report | Mexico | *Cal* |
| 2021 | Mothé *et al.* | Ocular lesions in a domestic feline: a closer look at the fungal pathogen *Sporothrix brasiliensis* | Brazil | *Cal* |
| 2022 | Almeida-Silva *et al.* | Beyond Domestic Cats: Environmental Detection of *Sporothrix brasiliensis* DNA in a Hyperendemic Area of Sporotrichosis in Rio de Janeiro State, Brazil | Brazil | *Nested-PCR* |
| 2022 | Rabello *et al.* | Environmental Isolation of *Sporothrix brasiliensis* in an Area With Recurrent Feline Sporotrichosis Cases | Brazil | *Beta, Cal, ITS, CHS* |
| 2022 | Gallo *et al.* | First three cases of cat-associated zoonotic cutaneous sporotrichosis in Colombia | Colombia | *Cal* |
| 2022 | Boechat *et al.* | *Sporothrix brasiliensis* and Feline Sporotrichosis in the Metropolitan Region of Rio de Janeiro, Brazil (1998-2018) | Brazil | *T3B* |
| 2022 | Queiroz-Telles *et al.* | Cutaneous disseminated sporotrichosis in immunocompetent patient: Case report and literature review | Brazil | *ITS, Cal* |
| 2022 | Andrade *et al.* | Household outbreak of sporotrichosis: towards the One Health approach | Brazil | *Cal* |
| 2022 | Rabello *et al.* | Preservation Methods in Isolates of *Sporothrix* Characterized by Polyphasic Approach | Brazil | *Beta* |
| 2022 | Rachman *et al.* | Zoonotic acquisition of cutaneous Sporothrix braziliensis infection in the UK. | United Kingdom | *ITS* |
| 2023 | Aguiar *et al.* | First case report of feline sporotrichosis caused by Sporothrix brasiliensis in the state of Ceará – Brazil. | Brazil | *ITS* |
| 2023 | Angelo *et al*. | Sporothrix brasiliensis infecting cats in northeastern Brazil: New emerging areas in Paraíba state. | Brazil | *Cal* |
| 2023 | Barnacle *et al.* | The first three reported cases of *Sporothrix brasiliensis* cat-transmitted sporotrichosis outside South America | England | *Beta* |
| 2023 | Chieosilapatham *et al*. | Sporothrix schenckii sensu stricto related to zoonotic transmission in Thailand. | Thailand | *Cal* |
| 2023 | Colombo et al. | Emergence of zoonotic sporotrichosis due to Sporothrix brasiliensis in Minas Gerais, Brazil: A molecular approach to the current animal disease | Brazil | *ITS* |
| 2023 | Escobar et al. | Sporothrix brasiliensis in cats from Santiago, Chile. | Chile | *ITS* |
| 2023 | Prado et al. | First Cases of Feline Sporotrichosis Caused by Sporothrix brasiliensis in Paraguay | Paraguay | *Cal* |
| 2023 | Thomson et al. | Sporotrichosis Outbreak Due to Sporothrix brasiliensis in Domestic Cats in Magallanes, Chile: A One-Health-Approach Study | Chile | *ITS* |
| 2023 | Waller et al. | Are γ-terpinene, 1,8-cineole, p-coumaric acid, and quercetin active against wild-type and non–wild-type Sporothrix brasiliensis to itraconazole? | Brazil | *PCR-RFLP* |
| 2023 | Yeow *et al*. | Mucosal Sporotrichosis from Zoonotic Transmission: Descriptions of Four Case Reports | Malasya | *Maldi-Tof* |
| 2023 | Yingchanakiat *et al.* | Phenotypic and Genotypic Characterization and Antifungal Susceptibility of Sporothrix schenckii sensu stricto Isolated from a Feline Sporotrichosis Outbreak in Bangkok, Thailand. | Thailand | *Cal; Beta* |

* Based in the first author affiliation

**References:**

1. Aguiar, B.A., Borges, I.S., Silva, B.W.L., Rodrigues, F.R.N., Gonçalves, L.D., Casseb, A.R., et al. (2023). First case report of feline sporotrichosis caused by Sporothrix brasiliensis in the state of Ceará – Brazil. *Med Mycol Case Rep,* [*S. l.*], v. 16, n. 40, p. 12-15. doi: 10.1016/j.mmcr.2023.02.005.
2. Almeida-Paes, R., Oliveira, M.M.E., Freitas, D.F.S., Valle, A.C.F., Gutierrez-Galhardo, M.C., Zancopé-Oliveira, R. (2016). Refractory sporotrichosis due to *Sporothrix* *brasiliensis* in humans appears to be unrelated to in vivo resistance. *Medical Mycology,* [*S. l.*], v. 55, n. 5., p. 507-517. DOI: 10.1093/mmy/myw103
3. Almeida-Paes, R., Oliveira, M.M.E., Freitas, DF.S., Valle, A.C.F., Zancopé-Oliveira, R.M., Gutierrez-Galhardo, M.C. (2014). Sporotrichosis in Rio de Janeiro, Brazil: *Sporothrix* *brasiliensis* Is Associated with Atypical Clinical Presentations. *PLoS Neglected Tropical Diseases*, [*S. l.*], v. 8, n. 9, p. e3094. DOI: 10.1371/journal.pntd.0003094
4. Almeida-Silva, F., Rabello, V.B.S., Scramignon-Costa, B.S., Zancopé-Oliveira, R.M., Macedo, P.M., Almeida-Paes, R. (2022). Beyond Domestic Cats: Environmental Detection of *Sporothrix* *brasiliensis* DNA in a Hyperendemic Area of Sporotrichosis in Rio de Janeiro State, Brazil. *J Fungi (Basel),* [S. l.], v. 8, n. 6, p. 604. doi: 10.3390/jof8060604.
5. Andrade, E.H.P., Bastos, C.V., Silva, A.V., Moreira, S.M., Costa, T.G.A., Salvato, L.A., et al. (2022). Household outbreak of sporotrichosis: towards the One Health approach. *Rev Soc Bras Med Trop,* [S. l.], v. 6, n. 55. doi: 10.1590/0037-8682-0021
6. Angelo, D. F. dos S., Rabello, V. B. de S., Maciel, M. A. S., Atanázio, S. S. de L. A., Costa, M. C. L. da ., Silva, S. R., Almeida-Paes, R., Bernardes-Engemann, A. R., Zancopé-Oliveira, R. M., & Clementino, I. J.. (2023). *Sporothrix brasiliensis* infecting cats in northeastern Brazil: New emerging areas in Paraíba state. *Ciência Rural*, *53*(10), e20220351. https://doi.org/10.1590/0103-8478cr20220351
7. Araujo, M. L., Rodrigues, A. M., Fernandes, G.F., Camargo, Hoog, G.S. (2015). Human sporotrichosis beyond the epidemic front reveals classical transmission types in Espírito Santo, Brazil. *MMMycoses,* [*S. l.*], v. 58, p. 485-490. DOI: 10.1111/myc.12346
8. Araújo, M.J.C.L.N., Nihei, C.H., Rodrigues, A.M., Higashino, H., Ponzio, V., Pignatari, A.C.C., et al. (2021). Case Report: Invasive Sinusitis due to *Sporothrix brasiliensis* in a Renal Transplant Recipient. *Am J Trop Med Hyg,* [S. l.], v. 105, n. 5, p.1218-1221. doi: 10.4269/ajtmh.20-1602.
9. Arechavala, A., Orduna, T., Maiolo, E., Mujica, M.T., Fernandez, M., Negroni, R. (2011). Esporotricosis diseminada con compromiso cutáneo y visceral. *Revista de patologia tropical*, [*S. l.*], v.40, n. 1, p. 73-84.
10. Azam, N.K.K., Selvarajah, G.T., Santhanam, J., Razak, M.F.A., Ginsapu, S.J., James, J.E. Suetrong, S. (2020). Molecular epidemiology of *Sporothrix schenkii* isolates in Malaysia. *Med Mycol*, [S. l.], v. 58, n. 5. p. 617-625. doi: 10.1093/mmy/myz106.
11. Alves, M.M., Milan, E.P., Silva-Rocha, W.P., Costa, A.S.S.P., Maciel, B.A., Vale, PHC., et al. (2020). Fatal pulmonary sporotrichosis caused by *Sporothrix brasiliensis* in Northeast Brazil. *PLoS Negl Trop Dis,* [S. l.], v. 14, n. 5. DOI: 10.1371/journal.pntd.0008141
12. Barnacle, J., Chow, Y.J., Borman, A.M., Wyllie, S., Dominguez, V., Russell, K. (2023). The first three reported cases of *Sporothrix brasiliensis* cat-transmitted sporotrichosis outside South America. *Med Mycol Case Rep,* [S. l.], v. 20, n. 39, p.14-17. doi: 10.1016/j.mmcr.2022.12.004.
13. Barreto, L., Velásquez, G., Mendoza, M., Camacho, E., Goncalves, E Rodríguez, S., Niño-Veja, G.A. (2021). Geographical distribution and ecological niche modeling of the etiological agents of human sporotrichosis in Venezuela. *Braz J Microbiol*, [S. l.], v. 52, n. 1, p. 63-71. doi: 10.1007/s42770-020-00306-0.
14. Boechat, J.S., Oliveira, M.M.E., Almeida-Paes, R., Gremiao, I.D.F., Machado, A.C.S., Oliveira, R.V.C., et al., (2018). Feline sporotrichosis: associations between clinical-epidemiological profiles and phenotypic-genotypic characteristics of the etiological agents in the Rio de Janeiro epizootic area. *Mem. Inst. Oswaldo Cruz*, [S. l.], v. 113, n. 3, p. 185–196. DOI: 10.1590/0074-02760170407.
15. Boechat, J.S., Oliveira, M.M.E., Almeida-Paes, R., Gremião, I.D.F., Machado, A.C.S., Oliveira, R.V.C., Figueiredo, A.B.F., Rabello, V.B.S., Silva, K.B.L., Zancopé-Oliveira, R.M., Schubach, T.M.P., Pereira, S.A. (2018). Feline sporotrichosis: associations between clinical-epidemiological profiles and phenotypic-genotypic characteristics of the etiological agents in the Rio de Janeiro epizootic area. *Memorias do Instituto Oswaldo Cruz*, [S. l.], v. 113, n. 3, p. 185–196. DOI: 10.1590/0074-02760170407
16. Boechat, J.S., Oliveira, M.M.E., Gremião, I.D.F., Almeida-Paes, R., Machado, A.C., Zancopé-Oliveira, R., et al. (2022). *Sporothrix brasiliensis* and Feline Sporotrichosis in the Metropolitan Region of Rio de Janeiro, Brazil (1998-2018). *J Fungi (Basel),* [S. l.], v. 8, n. 7, p. 749. doi: 10.3390/jof8070749.
17. Boechat, J.S., Pereira, S.A., Machado, A.C.S., Viana, P.G., Almeida-Paes, R., Zancopé-Oliveira, R.M., Gremião, I.D.F., Oliveira, M.M.E. (2021). Canine sporotrichosis: polyphasic taxonomy and antifungal susceptibility profiles of *Sporothrix* species in an endemic area in Brazil. Brazilian Journal of Microbiology: [publication of the Brazilian Society for Microbiology], [S. l.], v. 52, n. 1, p. 135–143. DOI: 10.1007/s42770-020-00328-8
18. Bonifaz, A., Morales-Peña, N., Tirado-Sánchez, A., Jiménez-Mendoza, D.R., Treviño-Rangel, R.J., González, G.M. (2020). Atypical sporotrichosis related to *Sporothrix mexicana.* *Mycopathologia,* [S. l.], v. 185, n. 4, p. 733-735. doi: 10.1007/s11046-020-00463-8.
19. Borba-Santos, L.P., Rodrigues, A.M., Gagini, T.B., Fernandes, G.F., Castro, R., Camargo, Z.P. et al. (2015). Susceptibility of *Sporothrix* *brasiliensis* isolates to amphotericin B, azoles, and terbinafine. *Medical Mycology*, [*S. l.*], v. 53, n. 2, p.178-188. DOI: 10.1093/mmy/myu056
20. Camacho, E., Léon-Navarro, I., Rodrígues-Brito, S., Mendoza, M., Nino-Veja, G. (2015). Molecular epidemiology of human sporotrichosis in Venezuela reveals high frequency of *Sporothrix* *globosa*. *BMC Infectious Diseases*, [*S. l.*], v. 15, n. 94. DOI: 10.1186/s12879-015-0839-6
21. Castro, R.A., Kubistschek, P.H., Teixeira, P.A.C., Sanches, G.F., Teixeira, M.M., Quintella, L.P. et al. (2013). Differences in cell morphometry, cell wall topography and gp70 expression correlate with the virulence of *Sporothrix* *brasiliensis* clinical isolates. *Plos One*, [*S. l.*], v. 8, n. 10. DOI: 10.1371/journal.pone.0075656
22. Caballero, A.A., Negrete, A., Guillermo J., Brunelli, P.J. Value of molecular techniques and risk factors in the diagnosis and evolution of sporotrichosis. About 2 cases of Sporothrix brasiliensis and S. globosa [A*n. Fac. Cienc. Méd*. (Asunción)](http://portal.revistas.bvs.br/transf.php?xsl=xsl/titles.xsl&xml=http://catserver.bireme.br/cgi-bin/wxis1660.exe/?IsisScript=../cgi-bin/catrevistas/catrevistas.xis|database_name=TITLES|list_type=title|cat_name=ALL|from=1|count=50&lang=pt&comefrom=home&home=false&task=show_magazines&request_made_adv_search=false&lang=pt&show_adv_search=false&help_file=/help_pt.htm&connector=ET&search_exp=An.%20Fac.%20Cienc.%20M%C3%A9d.%20(Asunci%C3%B3n)); 53(3): 177-184, 20201201.
23. Chieosilapatham, P., Chuamanochan, P., Chiewchavit, S., Saikruatep, R., Amornrungsun, E., Preechasuth, K. (2023). *Sporothrix schenckii* sensu stricto related to zoonotic transmission in Thailand. *Med Mycol Case Rep,* [*S. l.*], v. 18, n. 41, p. 44-47. doi: 10.1016/j.mmcr.2023.08.003.
24. Choappa, R.M.C., Oyarzo, P.I.V., Silva, L.C.C. (2014). Aislamiento de *Sporothrix* *pallida* complex en muestras clínicas y ambientales de Chile. *Revista Argentina de Microbiología*, [*S. l.*], v. 46, n. 4.
25. Colombo, S.A., Bicalho, G.C., Oliveira, C.S.F., Soares, D.F.M., Salvato, L.A., Keller, K.M., et al. (2023). Emergence of zoonotic sporotrichosis due to *Sporothrix brasiliensis* in Minas Gerais, Brazil: A molecular approach to the current animal disease. *Mycoses,* [*S. l.*], v. 66 n. 10, p. 911-922. doi: 10.1111/myc.13631.
26. Córdoba, S., Isla, G., Szusz, W., Vivot, W., Hevia, A., Davel, G., Canteros, G.E. (2018). Molecular identification and susceptibility profile of *Sporothrix schenckii* sensu lato isolated in Argentina. *Mycoses,* *[S.l.],* v. 61, n. 7. p. 441-448. doi: 10.1111/myc.12760.
27. De Beer, Z.W., Duong, T.A., Wingfield, M.J. (2016). The divorce of *Sporothrix* and Ophiostoma: solution to a problematic relationship. *Studies in Mycology*, South Africa, v. 83, p. 165–191. DOI: 10.1016/j.simyco.2016.07.001
28. De Meyer, E. M., De Beer, Z.W., Summerbell, R.C., Moharram, A.M., Hoog, G.S., Vismer, H.F., Wingfield, M.J. (2008). Taxonomy and phylogeny of new wood- and soil-inhabiting *Sporothrix* species in the Ophiostoma stenoceras - *Sporothrix schenckii* complex. *Mycologia*, [*S. l.*], v. 100, n. 4, p. 647-661. DOI: 10.3852/07-157r
29. Dias, N. M., Oliveira, M.M.E., Santos, C., Zancopé-Oliveira, R., Lima, N. (2011). Sporotrichosis Caused by *Sporothrix mexicana*, Portugal. *Emerg Infect Dis*, [*S. l.*], v. 17, n. 10, p. 1975-1976, 2. DOI: 10.3201/eid1710.110737
30. Errasti, A., de Beer, Z. W., Coetzee, M. P. A., Roux, J., Rajchenberg, M., Wingfield, M. J. (2016). Three new species of Ophiostomatales from Nothofagus in Patagonia. *Mycological Progress*, *[S. l.*], v. 15, n. 17.
31. Escobar, M.C., Ramos, F.C., Rojas, C.A.A. (2023). *Sporothrix brasiliensis* in cats from Santiago, Chile. *Med Mycol Case Rep*, [*S. l.*], v. 26, n. 43, p. 100624. doi: 10.1016/j.mmcr.2023.100624.
32. Eyer-Silva, W.A., Azevedo, M.C.V.M., Silva, G.A.R., Basílio-de-Oliveira, R.P., Araujo, L.F., Lago, I.V., et al. (2018). Palate ulcer, uvular destruction and nasal septal perforation caused by *Sporothrix brasiliensis* in an HIV-infected patient. *Med Mycol Case Rep*, [S. l.], v. 12, n. 23, p. 16-19. doi: 10.1016/j.mmcr.2018.11.001.
33. Fernandes, B., Caligiorne, R.B., Coutinho, D.M., Gomes, R.R., Rocha-Silva, F., Machado, A.S., *et al*. (2018). A case of disseminated sporotrichosis caused by *Sporothrix brasiliensis.* *Med Mycol Case Rep, [S.l.],* v. 21, n. 21. p. 34-36. doi: 10.1016/j.mmcr.2018.03.006.
34. Fernandes, G.F., Santos, P.O., Rodrigues, A.M., Sasaki, A.A., Burger, E., Camargo, Z.P. (2013). Characterization of virulence profile, protein secretion and immunogenicity of different *Sporothrix* *schenckii* sensu stricto isolates compared with *S. globosa* and *S. brasiliensis* species. *Virulence,* v. 4, n. 3. doi: 10.4161/viru.23112.
35. Fichman, V., Valle, A.C.F., Macedo, P.M.M, Freitas, D.F.S., Oliveira, M.M.E., Almeida-Paes, M., Gutierrez-Galhardo, M.C. (2018). Cryosurgery for the treatment of cutaneous sporotrichosis in four pregnant women
36. Fichman, V., Macedo, P.M., Freitas, D.F.S., Valle, A.C.F., Almeida-Silva, F., 1, Bernardes-Engemann, A.R. 2, et al. (2020). Zoonotic sporotrichosis in renal transplant recipients from Rio de Janeiro, Brazil. *Transpl Infect Dis,* [S. l.], v. 23, n. 2. doi: 10.1111/tid.13485.
37. Filho, J.E., Santos, I.B., Reis, C.M.S, Patané, J.S.L., Paredes, V., Bernardes, J.P.R.A., et al. (2020). A novel *Sporothrix brasiliensis* genomic variant in Midwestern Brazil: evidence for an older and wider sporotrichosis epidemic. *Emerg Microbes Infect,* [S. l.], v. 9, n. 2, p. 2515-2525. doi: 10.1080/22221751.2020.1847001.
38. Flórez-Muñoz, S.V., Alzate, J.F., Mesa-Arango, A.C. (2019). Molecular Identification and Antifungal Susceptibility of Clinical Isolates of *Sporothrix schenckii* Complex in Medellin, Colombia. *Mycopathologia,* [S. l.], v. 184, n. 1, p. 53-63. doi: 10.1007/s11046-018-0310-5.
39. Freitas, D.F., Santos, S.S., Almeida-Paes, R., Oliveira, M.M.E., Valle, A.C.F., Gutierrez-Galhardo, M.C. et al. (2015). Increase in virulence of *Sporothrix* *brasiliensis* over five years in a patient with chronic disseminated sporotrichosis. *Virulence*, [*S. l.*], v. 6, n. 2, p. 112-120. DOI: 10.1080/21505594.2015.1014274
40. Gallo, S., Arias-Rodriguez, C., Sánchez-Cifuentes, E.A., Santa-Vélez 1, Isabel Larrañaga-Piñeres, I., Gaviria-Barrera, M.E., et al. (2022). First three cases of cat-associated zoonotic cutaneous sporotrichosis in Colombia. *Int J Dermatol,* [S. l.], v. 61, n. 10, p. 1276-1279. doi: 10.1111/ijd.16377.
41. Gomez, O.M., Alvarez, L.C., Muñoz, A.J., Misas, E., Gallo, E.J., Jimenez, M.D.P., Arango, M., et al. (2018). Draft Genome Sequences of Two *Sporothrix schenckii* Clinical Isolates Associated with Human Sporotrichosis in Colombia. *Genome Announc,* [S. l.], v. 14, n. 6, p. 24. doi: 10.1128/genomeA.00495-18.
42. Gompertz, O.F., Rodrigues, A.M., Fernandes, G.F., Bentubo, Henri D L, Camargo, Zoilo Pires, Petri, Valéria. (2016). Atypical Clinical Presentation of Sporotrichosis Caused by *Sporothrix globosa* Resistant to Itraconazole. *Am J Trop Med Hyg,* [*S. l.*], v. 94, n. 6., p. 1218-22. DOI: 10.4269/ajtmh.15-0267.
43. Gong, J., Zhang, M., Wang, Y., Li, R., He, L., Wan, Z., Li, F., Zhang, J. (2019). Population Structure and Genetic Diversity of *Sporothrix globosa* in China According to 10 Novel Microsatellite Loci. *J Med Microbiol,* [S. l.], v. 68, n. 2, p. 248-254. doi: 10.1099/jmm.0.000896
44. Govender N.P., Maphanga T.G., Zulu T.G., Patel J., Walaza S., Jacobs C., Ebonwu, J.I., Ntuli, S., Naicker, S.D., Thomas, J. (2015). An outbreak of lymphocutaneous sporotrichosis among mine-workers in South Africa. *PLoS Neglected Tropical Diseases,* [*S. l.*], v. 9, n. 9., p. 40-96. DOI: 10.1371/journal.pntd.0004096
45. Gu, A., Zhang, X., Ma, F., Nie, Z., Hoog, G.S., Zhang, Y. (2020). Fixed cutaneous sporotrichosis in a patient with numerous fungal elements. *Med Mycol Case Rep,* [S. l.], v. 11, n. 29, p. 32-34. doi: 10.1016/j.mmcr.2020.06.001
46. Galati, L.H., Tomimori, J., Taborda, C.P., Michalany, N.S., Junior, C.E., & Larsson, C.E. (2017). Sporotrichosis – cat as the source of familiar infection outbreak in Guarulhos, São Paulo: a case report. *Brazilian Journal of Veterinary Research and Animal Science, 54*, 439-444.
47. Takazawa M, Harada K, Kakurai M, Yamada T, Umemoto N, Sakai T, Maeda T, Kawase M, Demitsu T. (2018). Case of pyoderma gangrenosum-like sporotrichosis caused by Sporothrix globosa in a patient with ulcerative colitis. *J Dermatol*. Aug;45(8):e226-e227. doi: 10.1111/1346-8138.14276.
48. Hu, Q., He, Y., Zhou, X. (2015). Construction and analysis of the cDNA subtraction library of yeast and mycelial phases of *Sporothrix globosa* isolated in China: identification of differentially expressed genes. *J Zhejiang Univ Sci B,* [*S. l.*], *v.*  16, n. 12, p. 991-7. DOI: 10.1631/jzus.B1500151.
49. Kano, R., Anzawa, K., Mochizuki, T., Nishimoto, K., Hiruma, M., Kamata, H., Hsegawa, A. (2013). *Sporothrix schenckii* (sensu strict *S. globosa*) mating type 1-2 (MAT1-2) gene. *The Journal of Dermatology*, [*S. l.*], v. 40, n. 9. DOI: 10.1111/1346-8138.12226
50. Kano, R., Okubo, M., Siew, H.H., Kamata, H., Hasegawa, A., (2015) Molecular typing of *Sporothrix schenckii* isolates from cats in Malaysia. *Mycoses,* [S. l.], v. 58, N. 4, P. 220–224. DOI: org/10.1111/myc.12302
51. Kano, R., Tsui, C.K.M., Hamelin, R.C., Anzawa, K., Mochizuki, T., Nishimoto, K., et al. (2015). The Mat1-1:Mat1-2 ratio of *Sporothrix* *globosa* isolates in Japan. *Mycopathologia,* [*S. l.*], v. 179, p. 81–86. Doi: 10.1007/s11046-014-9808-7
52. Kim, H.R., Shin, D.H., Lee, J., Choi, J.S. (2019). Molecular Identification of Human Sporotrichosis in Korea. *J Mycol Infc*, [S. l.], v. 24, n. 2, p. 45-51 doi: 10.17966/JMI.2019.24.2.45
53. Liu, F., Yingmei, L., Liu, Yuan, N., Zhang, X., Cao, M., Dong, J., Zhang, J. (2021). Fixed Cutaneous Sporotrichosis Due to *Sporothrix globosa.* *Clin Cosmet Investig Dermatol,* [S. l.], v. 25, n. 14, p. *91-96.* DOI: 10.2147/CCID.S288259.
54. Liu, T.; Zhang, K.; Zhou X. (2014). Molecular identification of *Sporothrix* clinical isolates in China. *J Zhejiang Univ-Sci B (Biomed & Biotechnol),* [*S. l.*], v. 15, n. 1, p. 100-108.
55. Macedo, P.M., Sztajnbok, D.C.N., Camargo, Z.P., Rodrigues, A.M., Lopes-Bezerra, L.M., Bernardes-Engemann, A.R., Orofino-Costa, R. (2015) Dacryocystitis due to *Sporothrix brasiliensis*: a case report of a successful clinical and serological outcome with low-dose potassium iodide treatment and oculoplastic surgery. *British Journal of Dermatology*, [*S. l.*], v. 172, n. 4. DOI: 10.1111/bjd.13378
56. Macêdo-Sales, P.A., Souza, L.O.P., Della-Terra, P.P., Lozoya-Pérez, N.E., Machado, R.L.D., Rocha, E.L.M.S. (2020). Coinfection of domestic felines by distinct *Sporothrix brasiliensis* in the Brazilian sporotrichosis hyperendemic área. *Fungal Genet Biol,* [S. l.], v. 140, n. 103397. doi: 10.1016/j.fgb.2020.103397.
57. Madrid, H., Cano, J., Gené, J., Bonifaz, A., Toriello, C., Guarro, J. (2009). *Sporothrix globosa*, a pathogenic fungus with widespread geographical distribution. *Revista Iberoamericana de Micología*, [*S. l.*], v. 26, n. 3, p. 218–222. DOI: 10.1016/j.riam.2009.02.005
58. Madrid, H., Gené, J., Cano, J., Silvera, C., Guarro, J. (2010) *Sporothrix* *brunneoviolacea* and *Sporothrix* *dimorphospora*, two new members of the Ophiostoma stenoceras-Sporothrix schenckii complex. *Mycologia*, [*S. l.*], v. 102, n. 5, p. 193-203.
59. Mahmoudi, S., Zaini, F., Kordbacheh, P., Safara, M., Heidari, M. (2016). *Sporothrix* *schenckii* complex in Iran: Molecular identification and antifungal susceptibility. *Medical Mycology,* [*S. l.*], v. 54, n. 6., p. 593-602. DOI: 10.1093/mmy/myw006
60. Makri, N., Paterson, G. K., Gregge, F. Urquhart, C., Nuttall, T. (2020). First case report of cutaneous sporotrichosis (*Sporothrix* species) in a cat in the UK. *JFMS Open Reports*, [S. l.], v. 6, n. 1, p. 20551169 -2090600. DOI: 10.1177/2055116920906001
61. Marimon, R., Cano, J. Gené, J., Sutton, D. A., Kawasaki, M., Guarro, J. (2007). *Sporothrix* *brasiliensis, S. globosa*, and *S. mexicana,* three new *Sporothrix* species of clinical interest. *Journal Clinical Microbiology*, [S. l.], v. 45, n. 10, p. 3198–3206. DOI: 10.1128/JCM.00808-07
62. Marimon, R., Gené, J., Cano, J., Guarro, J. (2008). *Sporothrix luriei*: a rare fungus from clinical origin. *Medical Mycology*, Spain, v. 46, n. 6, p. 621–625. DOI: 10.1080/13693780801992837
63. Martínez-Herrera, E., Arenas, R., Hernández-Castro, R., Frías-De-León, M.G., Rodríguez-Cerdeira, C. (2021). Uncommon Clinical Presentations of Sporotrichosis: A Two-Case Report. *Pathogens,* [S. l.], v. 10, n. 10. doi: 10.3390/pathogens10101249.
64. Matos, A.M.F., Moreira, M.L., Barczewski, B.F., Matos, L.X., Oliveira, J.B.V., Pimentel, M.I.F., et al. (2019). Identification by MALDI-TOF MS of *Sporothrix brasiliensis* Isolated from a Subconjunctival Infiltrative Lesion in an Immunocompetent Patient. *Microorganisms,* [S. l.], v. 8, n. 1, p. 22. doi: 10.3390/microorganisms8010022.
65. Mothé, B.B., Reis, N.F., Melivilu, C.S.I Junior, A.G.F.M., Santos, C.S., Dieckmann, A.M., Machado, R.L.D., et al. (2021). Ocular lesions in a domestic feline: a closer look at the fungal pathogen *Sporothrix brasiliensis. Braz J Vet Res Anim Sci.* [S. l.], v. 58. DOI: 10.11606/issn.1678-4456.bjvras.2021.183219
66. Musvuugwa, T., Beer, Z.W., Duong, T., Dreyer, L., Oberlander, K., Roets, F. (2016). Wounds on Rapanea melanophloeos provide habitat for a large diversity of Ophiostomatales including four new species. *Antonie Van Leeuwenhoek,* [*S. l.*], v. 109, n. 6. P. 877-894. DOI: 10.1007/s10482-016-0687-4
67. Musvuugwa, T., Beer, Z.W., Duong, T.A., Dreyer, L.L., Oberlander, K.O., Roets, F. (2015). New species of Ophiostomatales from Scolytinae and Platypodinae beetles in the Cape Floristic Region, including the discovery of the sexual state of Raffaelea. *Antonie Van Leeuwenhoek,* [*S. l.*], v. 108, n. 4., p. 933-950. DOI: 10.1007/s10482-015-0547-7
68. Nath, R., Lahon, P., Timung, L. (2017). Molecular identification and phenotypic characterisation of *Sporothrix globosa* from clinical cases of Eastern Assam, North-east India. *Indian J Med Microbiol*, *[S.l.],* v. 35, n. 2. p. 269-273. doi: 10.4103/ijmm.IJMM_16_264.
69. Nesseler, A., Schauerte, N., Geiger, C., Kaerger, K., Walther, G., Kurzai, O., Eisenberg, T. (2019). *Sporothrix humicola* (Ascomycota: Ophiostomatales) - A soil-borne fungus with pathogenic potential in the eastern quoll (Dasyurus viverrinus). *Medical Mycology Case Report*, [S.l.], v. 25, p. 39–44. DOI:10.1016/j.mmcr.2019.07.008.
70. Ochoa-Reyes, J., Ramos-Martínez, E., Treviño-Rangel, R., González, G.M., Bonifaz, A. (2018). Auricular sporotrichosis. Atypical case report simulating bacterial cellulitis. *Rev Chilena Infectol,* [S. l.], v. 35, n. 1. p. 83-87. doi: 10.4067/s0716-10182018000100083.
71. Oliveira, D. C., Lopes, P. G. M, Spader, T. B., Mahl, C. D., Tronco-Alves, G. R., Lara, V. M., Santurio, J.M., Alves, S.H. (2011). Antifungal Susceptibilities of *Sporothrix albicans, S. brasiliensis*, and *S. luriei* of the *S. schenckii* Complex Identified in Brazil. *Journal Clinical Microbiology*, [S. l.], v. 49, n. 8, p. 3047–3049. DOI: 10.1128/JCM.00255-11
72. Oliveira, M. M. E., Almeida-Paes, R., Muniz, M. M., Gutierrez-Galhardo, M. C., Zancope-Oliveira, R. M. (2011). Phenotypic and Molecular Identification of *Sporothrix* Isolates from an Epidemic Area of Sporotrichosis in Brazil. *Mycopathologia*, [S. l.], v. 172, n. 4, p. 257–267. DOI: 10.1007/s11046-011-9437-3
73. Oliveira, M. M. E., Almeida-Paes, R., Muniz, M.M., Barros, M.B.L., Galhardo, M.B.C., Zancopé-Oliveira, R. (2010). Sporotrichosis caused by *Sporothrix* *globosa* in Rio de Janeiro, Brazil: case report. *Mycopathologia*, [S. l.], v. 169, p. 359–363. DOI: 10.1007/s11046-010-9276-7
74. Oliveira, M. M. E., Maifrede, S.B., Ribeiro, M.A., Zancopé-Oliveira, R.M. (2013). Molecular identification of *Sporothrix* species involved in the first familial outbreak of sporotrichosis in the state of Espírito Santo, southeastern Brazil. *Mem Inst Oswaldo Cruz*, v. 108, n. 7, p. 936-938. DOI: 10.1590/0074-0276130239
75. Oliveira, M. M. E., Sampaio, P., Almeida-Paes, R., Pais, C., Gutierrez-Gualhardo, Zancope-Oliveira, R. (2012). Rapid Identification of *Sporothrix* Species by T3B Fingerprinting. *J Clin Microbiol*, v. 50, n. 6, p. 2159-2162. DOI: 10.1128/JCM.00450-12
76. Oliveira, M. M. E., Veríssimo, C., Sabino, R., Aranha, J., Zancopé-Oliveira, R.M., Sampaio, P. et al. (2014). First autochthone case of sporotrichosis by *Sporothrix* *globosa* in Portugal. *Diagn Microbiol Infect Dis*, [S.l.], v. 78, n. 4, p. 388-390. DOI: 10.1016/j.diagmicrobio.2013.08.023
77. Oliveira, M.M.E., Almeida-Paes, R., Corrêa-Moreira, D., Borba, C.M., Menezes, R.C., 1, Freitas, D.F.S., et al. (2019). A case of sporotrichosis caused by different *Sporothrix brasiliensis* strains: mycological, molecular, and virulence analyses. *Mem Inst Oswaldo Cruz,* [S. l.], v. 21, n. 114. doi: 10.1590/0074-02760190260.
78. Oliveira, M.M.E., Franco-Duarte, R., Romeo, O., Pais, C., Criseo, G., Sampaio, P., Zancope-Oliveira, R. M. (2015). Evaluation of T3B fingerprinting for identification of clinical and environmental *Sporothrix* species. *FEMS Microbiol Lett*, [S.l.], v. 362, p. 1-7. DOI: 10.1093/femsle/fnv027
79. Oliveira, M.M.E., Santos, C., Sampaio, P., Romeo, O., Aldeida-Paes, R., Pais, C. et al. (2015). Development and optimization of a new MALDI-TOF protocol for identification of the *Sporothrix* species complex. *Research in Microbiology,* [S.l.], v. 166, n. 2., p. 102-10. DOI: 10.1016/j.resmic.2014.12.008
80. Orofino-Costa, R., Unterstell, N., Gripp, A.C., Macedo, P.M., Brota, A., Dias, E. et al. (2013). Pulmonary cavitation and skin lesions mimicking tuberculosis in a HIV negative patient caused by *Sporothrix brasiliensis*. *Medical Mycology Case Report*, [*S. l.*], v. 2, p. 65-71. DOI: 10.1016/j.mmcr.2013.02.004
81. Prado, C.M., Razzolini, E., Santacruz, G., Ojeda, L., Geraldo, R.M., Segovia, N., Brunelli, J.P., Vicente, V.A., Svoboda, W.K., Queiroz-Telles, F. (2023). First Cases of Feline Sporotrichosis Caused by *Sporothrix brasiliensis* in Paraguay. *J Fungi,* v.9, n. 10, p. 972. doi: 10.3390/jof9100972.
82. Queiroz-Telles, F., Cognialli, R.C., Salvador, G.L., Moreira, G.A., Herkert, P.F., Hagen, F. (2022). Cutaneous disseminated sporotrichosis in immunocompetent patient: Case report and literature review. *Med Mycol Case Rep,* [S. l.], v. 8, n. 36, p. 31-34. doi: 10.1016/j.mmcr.2022.05.003.
83. Rabello, V.B.S., Almeida-Silva, F., Scramignon-Costa, B.S., Motta, B.S., Macedo, P.M., Teixeira, M.M., et al. (2022). Environmental Isolation of *Sporothrix brasiliensis* in an Area With Recurrent Feline Sporotrichosis Cases. *Front Cell Infect Microbiol,* [S. l.], v. 12, n. 12, p. 894297. doi: 10.3389/fcimb.2022.894297.
84. Rabello, V.B.S., Corrêa-Moreira, D., Santos, C., Pinto, T.C.A.B., Procopio-Azevedo, A.C., Boechat, J., et al. (2022). Preservation Methods in Isolates of *Sporothrix* Characterized by Polyphasic Approach. *J Fungi (Basel),* [S. l.], v. 9, n. 1, p. 34. doi: 10.3390/jof9010034.
85. Rachman, R., Ligaj, M., Chinthapalli, S., Wani, R.S. (2022). Zoonotic acquisition of cutaneous *Sporothrix braziliensis* infection in the UK. *BMJ Case Rep,* [*S. l.*], v. 15, n. 5. doi: 10.1136/bcr-2021-248418.
86. Ramírez-Soto, M.C., Aguilar-Ancori, E.G., Quispe-Ricalde, M.A., Muñiz-Duran, G.A., Quispe-Florez, M.M., Chinen, A., (2021). Molecular identification of *Sporothrix* species in a hyperendemic area in Peru. *J Infect Public Health,* [S. l.], v. 14, n. 5, p. 670-673. doi: 10.1016/j.jiph.2021.02.005
87. Ramírez, F.A., Herrera, A., Bahena, M.T., Barrera, A.A., Cedillos, C. A. M., Bonifaz, A. (2010). Esporotricosis cutánea fija en un adolescente causada por *Sporothrix schenckii* (sensu stricto) y revisión comparativa de la bibliografia. *Medicine*, [*S. l.*]. DOI: 85827285
88. Reinprayoon, U., Jermjutitham, M., Tirakunwichcha, S., Banlunara, W., Tulvatana, W., Chindamporn, A. (2020). Conjunctival sporotrichosis from cat to human: Case report. *Am J Ophthalmol Case Rep,* [S. l.], v. 20, n. 20. DOI: 10.1016/j.ajoc.2020.100898
89. Rodrigues A.M., Camargo, E.B.Z.P., Bosco, S.M.G. (2014). *Sporothrix schenckii* sensu stricto isolated from soil in an armadillo's burrow. *Mycopathologia.* [S. l.], v. 177, n. 3-4, p. 199-206. DOI 10.1007/s11046-014-9734-8.
90. Rodrigues, A. M., Choappa, R. C., Fernandes, G. F., Hoog G. S., Camargo Z. P. (2016). *Sporothrix chilensis* sp. nov. (Ascomycota: Ophiostomatales), a soil-borne agent of human sporotrichosis with mild-pathogenic potential to mammals. *Fungal Biology*. São Paulo, v. 120, n. 2, p. 246–264. DOI: 10.1016/j.funbio.2015.05.006
91. Rodrigues, A. M., De Hoog, G. S., De Camargo, Z. P. (2014). Genotyping species of the *Sporothrix schenckii* complex by PCR-RFLP of calmodulin. *Diagnostic Microbiology Infectious Diseases*., [S. l.], v. 78, n. 4, p. 383–387. DOI: 10.1016/j.diagmicrobio.2014.01.004
92. Rodrigues, A. M., De Hoog, S., De Camargo, Z. P. (2013b). Emergence of pathogenicity in the *Sporothrix schenckii* complex. *Medical Mycology*., São Paulo, v. 51, n. 4, p. 405–412. DOI: 10.3109/13693786.2012.719648
93. Rodrigues, A. M., Teixeira, M. M., Hoog, G. S., Schubach, T. M. P., Pereira, S. A., Feranndes, G. F., Bezerra, L.M.L., Felipe, M.S., Camargo, Z.P. (2013a). Phylogenetic analysis reveals a high prevalence of *Sporothrix brasiliensis* in feline sporotrichosis outbreaks. *PLoS Negl Trop Dis*, São Paulo, v. 7, n. 6, p. 2281. DOI: 10.1371/journal.pntd.0002281
94. Rodrigues, A.M., Hoog, G.S., Pires, D.C., Brilhante, R.S.N., Sidrim, J.J.C., Gadelha, M.F. et al. (2014). Genetic diversity and anti fungal susceptibility profiles in causative agents of sporotrichosis. *BMC Infectious Diseases*, [*S. l.*], v.14, n. 219. DOI: 10.1186/1471-2334-14-219
95. Rodrigues, A.M., Hoog, G.S., Zhang, Y., Camargo, Z.P. (2014). Emerging sporotrichosis is driven by clonal and recombinant *Sporothrix* species. *Emerging Microbes and Infections,* [*S. l.*], v. 3, n. 5. DOI: 10.1038/emi.2014.33
96. Rodrigues, A.M., Najafzadeh, M.J., Hoog, G.S., Camargo, Z.P. (2015). Rapid Identification of Emerging Human-Pathogenic *Sporothrix* Species with Rolling Circle Amplification. *Frontiers in Microbiology,* [*S. l.*], v. 6, n. 1385. DOI: 10.3389/fmicb.2015.01385
97. Rojas, O.C., Bonifaz, A., Campos, C., Treviño-Rangel, R.J., González-Álvarez, R., González, G.M. (2018). Molecular Identification, Antifungal Susceptibility, and Geographic Origin of Clinical Strains of *Sporothrix schenckii* Complex in Mexico. *J Fungi (Basel)*, [S. l.], v. 4, n. 3. p. 86.doi: 10.3390/jof4030086.
98. Romeo, O.; Scordino, F.; Criseo, G. (2011). New insight into molecular phylogeny and epidemiology of *Sporothrix schenckii* species complex based on calmodulin-encoding gene analysis of Italian isolates. *Mycopathologia*, v. 172, p. 179 – 186.
99. Rudramurthy, S.M., Shankarnarayan, S.A., Hemashetter, B.M., Verma, S., Chauhan, S., Nath, R., et al. (2021). Phenotypic and molecular characterisation of Sporothrix globosa of diverse origin from India. *Braz J Microbiol,* [S. l.], v. 52, n. 1, p. 91-100. doi: 10.1007/s42770-020-00346-6.
100. Rosamoelina, T., Maubon, D., Raharolahy, O., Razanakoto, H., Rakotozandrindrainy, N., Rakotomalala, F.A., et al. (2019). Sporotrichosis in the Highlands of Madagascar, 2013-2017. *Emerg Infect DiS*, [S. l.], v. 25, n. 10, p. 1893-1902. doi: 10.3201/eid2510.190700.
101. Sasak, A.A., Fernandes, G.F., Rodrigues, A.M., Lima, F.M., Marini, M.M., Feitosa, LF, Teixeira, M.M. et al. (2014). Chromosomal Polymorphism in the *Sporothrix* *schenckii* Complex. *PLOS ONE*, v. 9, n. 1. DOI: 10.1371/journal.pone.0086819
102. Sendrasoa, F.A., Ranaivo, I.M., Sata, M., 1, Razanakoto, N.H., Andrianarison, M., Ratovonjanahary, V., Raharolahy, O., et al. (2021). Osteoarticular sporotrichosis in an immunocompetent patient. *Med Mycol Case Rep,* [S. l.], v. 26, n. 32, p. 50-52. doi: 10.1016/j.mmcr.2021.03.007.
103. Silva-Vergara, M. L., Camargo, Z.P., Silva, P.F., Abdalla, M.R., Sgarbieri, R.N., Rodrigues, A.M., et al. (2012). Disseminated *Sporothrix brasiliensis* infection with endocardial and ocular involvement in an HIV-infected patient. *Am J Trop Med Hyg*, [*S. l.*], v. 86, p. 477-480. DOI: 10.4269/ajtmh.2012.11-0441
104. Spanamberg, A., Araujo, R., Ravazzolo, A.P., Driemeier, D., Driemeier, R.M.S., Ferreiro, L. (2021). *Sporothrix brasiliensis* on cats with skin ulcers in Southern Brazil. *Med Mycol*, [S. l.], v. 59, n. 3, p. 301-304. doi: 10.1093/mmy/myaa083.
105. Stopiglia, C.D.O., Magagnin, C.M., Castrllón, M.R., Mendes, S.D.C., Heidrich, D., Valente, P. et al. (2014). Antifungal susceptibilities and identification of species of the *Sporothrix* *schenckii* complex isolated in Brazil*. Medical Mycology*, [*S. l.*], v. 52, n. 1, p. 56-64. DOI: 10.3109/13693786.2013.818726
106. Suzuki, R., Yikelamu, A., Tanaka, R., Igawa, K., Yokozeki, H., Yaguchi, T. (2016). Studies in Phylogeny, Development of Rapid Identification Methods, Antifungal Susceptibility, and Growth Rates of Clinical Strains of *Sporothrix* *schenckii* Complex in Japan. *Medical Mycology Journal,* [*S.l.],* v. 57, n. 3. p. 47-57. DOI: 10.3314/mmj.16-00005
107. Teixeira, M.M., Almeida, L.G.P., Kubitschek-Barreira, P., Alez, F.L., Kioshima, E.S., Abadio, A.K.R. (2014). Comparative genomics of the major fungal agents of human and animal Sporotrichosis: *Sporothrix schenckii* and *Sporothrix brasiliensis* *BMC Genomics,* v. 15. DOI: 10.1186/1471-2164-15-943
108. Thomson, J., Trott, D.J., Malik, R., Galgut, B., McAllister, M.M., Nimmo, J., Renton, D., Kidd, S.E. (2019). An atypical cause of sporotrichosis in a cat. *Med Mycol Case Rep*, [S. l.], v. 16, n. 23, p. 72-76. doi: 10.1016/j.mmcr.2019.01.004.
109. Thomson, P., González, C., Blank, O., Ramírez, V., Del Río, C., Santibáñez, S., Pena, P. (2023). Sporotrichosis Outbreak Due to *Sporothrix brasiliensis* in Domestic Cats in Magallanes, Chile: A One-Health-Approach Study. *J Fungi (Basel),* [*S. l.*], v. 9, n. 2, p. 226. doi: 10.3390/jof9020226.
110. Valeriano, C. A. T. (2020). Is *Sporothrix chilensis* circulating outside Chile? *PLOS Neglected Tropical Diseases,* [*S. l.*], v. 14, n. 3, p. e0008151.
111. Vettorato, R., Heidrich, D., Fraga, R., Ribeiro, A.M., Pagani, D.M., Timotheo, C., Amaro, T.G., Vettorato, G., Scroferneker, M.L. Sporotrichosis by *Sporothrix schenckii* senso stricto with itraconazole resistance and terbinafine sensitivity observed in vitro and in vivo: Case report. (2017). *Med Mycol Case Rep*, *[S.l.],* v. 28, n. 19. p.18-20. doi: 10.1016/j.mmcr.2017.10.001.
112. Waller, S.B., Ripoll,M.K., Gonçalves, H.P., Lana, D.F.D., Faria, R.O., Meireles, M.C.A., Fuentefria, A.M. Mello, J.R.B., Cleff, M.R. (2023). Are γ-terpinene, 1,8-cineole, p-coumaric acid, and quercetin active against wild-type and non–wild-type *Sporothrix brasiliensis* to itraconazole? *Braz J Microbiol,* [*S. l.*], v. 54, n. 1, p. 531-541. doi: 10.1007/s42770-022-00879-y
113. Wang, R., Jing, H., Chen, S., Yang, Y., Nan, H., Chen,T. (2019). A Patient With Sporotrichosis Diagnosed By Molecular Biology Combined With Traditional Methods. *J Med Cases*, [S. l.], v. 10, n. 9, p. 284-287. doi: 10.14740/jmc3362.
114. Xirotagaros, G., Drogari-Apiranthitou, M., Panayiotides, I.G., Tsakiraki, Z., Tsamakis, C. Theotokoglou, S. et al. (2015). Imported lymphocutaneous sporotrichosis in Greece. *British Journal of Dermatology*, [*S. l.*], v. 173, n. 3, p. 291-293. DOI: 10.1111/bjd.13558
115. Yeow, Y.Y., Tan, X.T., Low, L.L. (2023). Mucosal Sporotrichosis from Zoonotic Transmission: Descriptions of Four Case Reports. *Infect Dis Re,* [*S. l.*], v. 15, n. 1, p.102-111. doi: 10.3390/idr15010011.
116. Yingchanakiat, K., Limsivilai, O., Sunpongsri, S., Niyomtham, W., Lugsomya, K., Yurayart, C. (2023). Phenotypic and Genotypic Characterization and Antifungal Susceptibility of *Sporothrix* *schenckii* sensu stricto Isolated from a Feline Sporotrichosis Outbreak in Bangkok, Thailand. *J Fungi (Basel),* [*S. l.*], v.9, n. 5., p. 590. doi: 10.3390/jof9050590.
117. Yu, X., Wan, Z., Zhang, Z., Li, F., Li, R. & Liu, X. (2013). Phenotypic and molecular identification of *Sporothrix* isolates of clinical origin in Northeast China. *Mycopathologia*, [*S. l.*], v. 176, p. 67–74.
118. Zhang, Y., Hagen, F., Stielow, B., Rodrigues, A.M., Samerpitak, K., Zhou, X., Fen, et al. (2015). Phylogeography and evolutionary patterns in *Sporothrix* spanning more than 14 000 human and animal case reports. *Persoonia*, [*S. l.*], v. 35, p. 1-20. DOI: 10.3767/003158515X687416
119. Zhang Y, Hagen F, Wan Z, Liu Y, Liu Y, Wang Q, de Hoog GS, Li R, Zhang J. (2016) .Two cases of sporotrichosis of the right upper extremity in right-handed patients with diabetes mellitus. *Rev Iberoam Micol*. Jan-Mar;33(1):38-42. doi: 10.1016/j.riam.2015.02.001.
120. Zhang, Z., Liu, X., Lv, X., Lin, J. (2011). Variation in genotype and higher virulence of a strain of *Sporothrix schenckii* causing disseminated cutaneous sporotrichosis. *Mycopathologia*, [*S. l.*], v. 172, p. 439-446. DOI: 10.1007/s11046-011-9441-7
121. Zhao, L., Cui, Y., Zhen, Y., Yao, Y., Shi, Y., Song, Y., Chen, R., Li, S. Genetic variation of *Sporothrix globosa* isolates from diverse geographic and clinical origins in China. (2017). *Emerg Microbes Infect*, *[S.l.],* v. 6, n. 10. p. 88. doi: 10.1038/emi.2017.75.
122. Zhou, X., Léon-Navrro, I., Rodrígues-Brito, S., Mendoza, M., Nino-Veja, G. (2014). Global ITS diversity in the *Sporothrix* *schenckii* complex. *Fungal Diversity*, v. 66, n. 1, p. 153-165. DOI: 10.1186/s12879-015-0839-6
