# Supplementary material for: Systematic review of literature to evaluate global distribution of species of the Sporothrix genus stored in culture collections
Source: Front Cell Infect Microbiol. 2024 May 17;14:1382508. doi: 10.3389/fcimb.2024.1382508 (PMC11140055; doi:10.3389/fcimb.2024.1382508)
Supplement: Supplementary file 2 [file Table_2.docx]

Supplementary Table 2: *Sporothrix* species described in the world from the period 2007 - 2023. Absolute number and percentages of the species stored in collections.

| **Country** | **Species** | **Clade** | **Strain origin** | | | **Strain in Collection** | | | | | | | **Percentage of strains in culture collections (%)** |
| --- | --- | --- | --- | --- | --- | --- | --- | --- | --- | --- | --- | --- | --- |
|  |  |  | HMN | ANM | ENV | ATCC | CBS | CMW | IFM | IHEM | MUM | IOC |  |
| NORTH AMERICA | | | | | | | | | | | | | |
| USA (n= 47) | *S. brunneoviolacea* | ENV | 0 | Ant - 1 | 1 | 0 | 1 | 0 | 0 | 0 | 0 | 0 | 50% (1) |
|  | *S. dimorphospora* | ENV | 0 | 0 | 1 | 0 | 1 | 0 | 0 | 0 | 0 | 0 | 100% (1) |
|  | *S. eucastanea* | ENV | 0 | 0 | 1 | 0 | 0 | 1 | 0 | 0 | 0 | 0 | 100% (1) |
|  | *S. globosa* | CLI | 7 | 0 | 0 | 0 | 1 | 0 | 0 | 0 | 0 | 0 | 14% (1) |
|  | *S. gossypina* | ENV | 0 | 0 | 1 | 0 | 0 | 1 | 0 | 0 | 0 | 0 | 100% (1) |
|  | *S. rossii* | ENV | 0 | Beetle -1 | 0 | 0 | 0 | 1 | 0 | 0 | 0 | 0 | 100% (1) |
|  | *S. schenckii* | CLI | 35 | 0 | 0 | 3 | 5 | 0 | 0 | 0 | 0 | 0 | 22% (8) |
| Canada (n= 1) | *S. dimorphospora* | ENV | 0 | 0 | 1 | 0 | 3 | 0 | 0 | 0 | 0 | 0 | 100% (1) |
| Mexico (n= 100) | *S. abietina* | ENV | 0 | 0 | 1 | 0 | 1 | 0 | 0 | 0 | 0 | 0 | 100% (1) |
|  | *S. mexicana* | ENV | 1 | 0 | 2 | 0 | 2 | 0 | 0 | 0 | 0 | 0 | 66% (2) |
|  | *S. schenckii* | CLI | 87 | 0 | 6 | 0 | 1 | 0 | 0 | 0 | 0 | 0 | 1% (1) |
|  | *S. brasiliensis* | CLI | 1 | 0 | 0 | 0 | 0 | 0 | 0 | 0 | 0 | 0 | 0% (0) |
|  | *S. globosa* | CLI | 3 | 0 | 0 | 0 | 0 | 0 | 0 | 0 | 0 | 0 | 0% (0) |
| CENTRAL AMERICA | | | | | | | | | | | | | |
| Costa Rica (n= 1) | *S. schenckii* | CLI | 1 | 0 | 0 | 0 | 0 | 0 | 0 | 0 | 0 | 0 | 0% (0) |
| Guatemala (n= 3) | *S. globosa* | CLI | 1 | 0 | 0 | 0 | 0 | 0 | 0 | 0 | 0 | 0 | 0% (0) |
|  | *S. schenckii* | CLI | 2 | 0 | 0 | 0 | 0 | 0 | 0 | 0 | 0 | 0 | 0% (0) |
| SOUTH AMERICA | | | | | | | | | | | | | |
| Argentina (n= 19) | *S. brasiliensis* | CLI | 2 | Cat -5 | 0 | 0 | 2 | 0 | 0 | 0 | 0 | 0 | 35% (2) |
|  | *S. cabralli* | ENV | 0 | 0 | 3 | 0 | 0 | 1 | 0 | 0 | 0 | 0 | 33% (1) |
|  | *S. epigloea* | ENV | 0 | 0 | 1 | 0 | 1 | 0 | 0 | 0 | 0 | 0 | 100% (1) |
|  | *S. lignivora* | ENV | 0 | 0 | 1 | 0 | 0 | 0 | 0 | 0 | 0 | 0 | 0% (0) |
|  | *S. schenckii* | CLI | 5 | Equine -1 Mouse - 1 | 0 | 0 | 1 | 0 | 0 | 0 | 0 | 0 | 14% (1) |
| Bolivia (n= 2) | *S. schenckii* | CLI | 2 | 0 | 0 | 0 | 1 | 0 | 0 | 1 | 0 | 0 | 100% (2) |
| Brazil (n= 814) | *O. stenocera* | ENV | 0 | 0 | 2 | 0 | 1 | 1 | 0 | 0 | 0 | 0 | 100% (2) |
|  | *S. brasiliensis* | CLI | 230 | Cat – 203  Dog -51 | 3 | 3 | 40 | 0 | 0 | 0 | 0 | 0 | 11% (43) |
|  | *S. globosa* | CLI | 34 | 0 | 0 | 0 | 13 | 0 | 0 | 2 | 0 | 0 | 38% (15) |
|  | *S. luriei* | CLI | 1 | Dog -1 | 0 | 0 | 1 | 0 | 0 | 0 | 0 | 0 | 50% (2) |
|  | *S. mexicana* | ENV | 4 | 0 | 0 | 0 | 4 | 0 | 0 | 0 | 0 | 0 | 100% (4) |
|  | *S. pallida* | ENV | 7 | Cat -2 | 0 | 0 | 0 | 0 | 0 | 0 | 0 | 0 | 0% (0) |
|  | *S. schenckii* | CLI | 253 | Cat - 2  Dog -2 | 11 | 6 | 21 | 0 | 0 | 0 | 0 | 3 | 10% (27) |
| Chile (n= 12) | *S. brasiliensis* | CLI | 0 | Cat- 4 | 0 | 0 | 0 | 0 | 0 | 0 | 0 | 0 | 0% (0) |
|  | *S. chilensis* | ENV | 1 | 0 | 1 | 0 | 2 | 0 | 0 | 0 | 0 | 0 | 100% (2) |
|  | *S. dimorphospora* | ENV | 0 | 0 | 3 | 0 | 3 | 0 | 0 | 0 | 0 | 0 | 100% (3) |
|  | *S. dombeyi* | ENV | 0 | 0 | 1 | 0 | 0 | 1 | 0 | 0 | 0 | 0 | 100% (1) |
|  | *S. inflata* | ENV | 0 | 0 | 1 | 0 | 1 | 0 | 0 | 0 | 0 | 0 | 100% (1) |
|  | *S. pallida* | ENV | 0 | 0 | 1 | 0 | 1 | 0 | 0 | 0 | 0 | 0 | 100% (1) |
| Colombia (n= 53) | *S. globosa* | CLI | 13 | 0 | 0 | 0 | 0 | 0 | 0 | 0 | 0 | 0 | 0% (0) |
|  | *S. schenckii* | CLI | 39 | Cat-1 | 0 | 0 | 2 | 0 | 0 | 3 | 0 | 0 | 13% (5) |
| Paraguay (n= 2) | *S. brasiliensis* | CLI | 0 | Cat -2 | 0 | 0 | 0 | 0 | 0 | 0 | 0 | 0 | 0% (0) |
| Peru (n= 32) | *S. schenckii* | CLI | 13 | 0 | 0 | 0 | 4 | 0 | 0 | 9 | 0 | 0 | 100% (13) |
| Venezuela (n= 85) | *S. globosa* | CLI | 10 | 0 | 0 | 0 | 0 | 0 | 0 | 0 | 0 | 0 | 0% (0) |
|  | *S. schenckii* | CLI | 70 | 0 | 5 | 0 | 0 | 0 | 0 | 0 | 0 | 0 | 0% (0) |
| EUROPE | | | | | | | | | | | | | |
| Austria (n= 10) | *S. brunneoviolacea* | ENV | 0 | 0 | 2 | 0 | 2 | 0 | 0 | 0 | 0 | 0 | 100% (2) |
|  | *S. lunatum* | ENV | 0 | 0 | 1 | 0 | 1 | 0 | 0 | 0 | 0 | 0 | 100% (1) |
|  | *S. pallida* | ENV | 0 | 0 | 7 | 0 | 0 | 0 | 0 | 0 | 0 | 0 | 0% (0) |
| United Kingdom (n= 8) | *S. brasiliensis* | CLI | 3 | Cat -1 | 0 | 0 | 0 | 0 | 0 | 0 | 0 | 0 | 0% (0) |
|  | *S. globosa* | ENV | 1 | 0 | 0 | 0 | 1 | 0 | 0 | 0 | 0 | 0 | 100% (1) |
|  | *S. pallida* | ENV | 0 | 0 | 2 | 0 | 1 | 1 | 0 | 0 | 0 | 0 | 100% (2) |
|  | *S. humicola* | ENV | 0 | Cat- 1 | 0 | 0 | 1 | 0 | 0 | 0 | 0 | 0 | 100% (1) |
|  | *S. brasiliensis* | CLI | 3 | Cat-1 | 0 | 0 | 0 | 0 | 0 | 0 | 0 | 0 | 0% (0) |
| France (n= 1) | *S. schenckii* | CLI | 1 | 0 | 0 | 0 | 1 | 0 | 0 | 0 | 0 | 0 | 100% (1) |
| Germany (n= 11) | *S. brunneoviolacea* | ENV | 0 | 0 | 1 | 0 | 1 | 0 | 0 | 0 | 0 | 0 | 100% (1) |
|  | *S. inflata* | ENV | 0 | 0 | 1 | 0 | 1 | 0 | 0 | 0 | 0 | 0 | 100% (1) |
|  | *S. nivea* | ENV | 0 | 0 | 1 | 0 | 1 | 0 | 0 | 0 | 0 | 0 | 100% (1) |
|  | *S. pallida* | ENV | 0 | Insect - 1 | 5 | 0 | 2 | 0 | 0 | 0 | 0 | 0 | 33% (2) |
| Greece (n= 1) | *S. schenckii* | CLI | 1 | 0 | 0 | 0 | 1 | 0 | 0 | 0 | 0 | 0 | 100% (1) |
| Hungary (n= 1) | *S. dentifunda* | ENV | 0 | 0 | 1 | 0 | 0 | 1 | 0 | 0 | 0 | 0 | 100% (1) |
| Italy (n= 14) | *S. globosa* | CLI | 1 | 0 | 0 | 0 | 0 | 0 | 0 | 1 | 0 | 0 | 100% (1) |
|  | *S. mexicana* | ENV | 0 | Dog - 1 | 0 | 0 | 1 | 0 | 0 | 0 | 0 | 0 | 100% (1) |
|  | *S. pallida* | ENV | 0 | 0 | 6 | 0 | 0 | 0 | 0 | 0 | 0 | 0 | 0% (0) |
|  | *S. schenckii* | CLI | 4 | 0 | 1 | 0 | 1 | 0 | 0 | 0 | 0 | 0 | 20% (1) |
| Netherlands (= 14) | *S. dimorphospora* | ENV | 0 | 0 | 2 | 0 | 2 | 0 | 0 | 0 | 0 | 0 | 100% (2) |
|  | *S. foliorum* | ENV | 0 | 0 | 1 | 0 | 1 | 0 | 0 | 0 | 0 | 0 | 100% (1) |
|  | *S. inflata* | ENV | 0 | 0 | 2 | 0 | 0 | 2 | 0 | 0 | 0 | 0 | 100% (2) |
|  | *S. narcissi* | ENV | 0 | 0 | 1 | 0 | 0 | 1 | 0 | 0 | 0 | 0 | 100% (1) |
|  | *S. pallida* | ENV | 1 | 0 | 4 | 0 | 2 | 0 | 0 | 0 | 0 | 0 | 40% (2) |
|  | *S. schenckii* | CLI | 2 | 0 | 0 | 0 | 1 | 0 | 0 | 0 | 0 | 0 | 50% (1) |
| Norway (n= 2) | *O. stenoceras* | ENV | 0 | 0 | 2 | 0 | 1 | 1 | 0 | 0 | 0 | 0 | 100% (2) |
| Poland (n= 1) | *S. polyporicola* | ENV | 0 | 0 | 1 | 0 | 0 | 1 | 0 | 0 | 0 | 0 | 100% (1) |
| Portugal (n= 2) | *S. globosa* | CLI | 1 | 0 | 0 | 0 | 0 | 0 | 0 | 0 | 0 | 0 | 0% (0) |
|  | *S. mexicana* | ENV | 1 | 0 | 0 | 0 | 0 | 0 | 0 | 0 | 0 | 1 | 100% (1) |
| Spain (n= 35) | *S. brunneoviolacea* | ENV | 0 | 0 | 4 | 0 | 4 | 0 | 0 | 0 | 0 | 0 | 100% (4) |
|  | *S. cantabriensis* | ENV | 0 | Insect -1 | 0 | 0 | 0 | 1 | 0 | 0 | 0 | 0 | 100% (1) |
|  | *S. dimorphospora* | ENV | 0 | 0 | 2 | 0 | 2 | 0 | 0 | 0 | 0 | 0 | 100% (2) |
|  | *S. euskadiensis* | ENV | 0 | Beetle - 1 | 0 | 0 | 0 | 1 | 0 | 0 | 0 | 0 | 100% (1) |
|  | *S. globosa* | CLI | 14 | 0 | 0 | 0 | 5 | 1 | 0 | 0 | 0 | 0 | 43% (6) |
|  | *S. nebularis* | ENV | 0 | Insect -1 | 0 | 0 | 0 | 1 | 0 | 0 | 0 | 0 | 100% (1) |
|  | *S. pallida* | ENV | 0 | 0 | 12 | 0 | 0 | 0 | 0 | 0 | 0 | 0 | 0% (0) |
| Sweeden (n= 3) | *O. stenoceras* | ENV | 0 | Weevil - 1 | 0 | 0 | 0 | 1 | 0 | 0 | 0 | 0 | 100% (1) |
|  | *S. inflata* | ENV | 0 | 0 | 1 | 0 | 1 | 0 | 0 | 0 | 0 | 0 | 100% (1) |
|  | *S. polyporicola* | ENV | 0 | 0 | 1 | 0 | 1 | 0 | 0 | 0 | 0 | 0 | 100% (1) |
| AFRICA | | | | | | | | | | | | | |
| Kenya (n= 1) | *O. stenoceras* | ENV | 0 | 0 | 1 | 0 | 0 | 1 | 0 | 0 | 0 | 0 | 100% (1) |
| Madagascar (n= 12) | *S. schenckii* | CLI | 12 | 0 | 0 | 0 | 0 | 0 | 0 | 0 | 0 | 0 | 0% (0) |
| Mozambique (n= 1) | *S. schenckii* | CLI | 1 | 0 | 0 | 0 | 1 | 0 | 0 | 0 | 0 | 0 | 100% (1) |
| South Africa (n= 95) | *S. aemulophila* | ENV | 0 | 0 | 4 | 0 | 0 | 4 | 0 | 0 | 0 | 0 | 100% (4) |
|  | *S. africana* | ENV | 0 | 0 | 1 | 0 | 0 | 1 | 0 | 0 | 0 | 0 | 100% (1) |
|  | *S. africanum* | ENV | 0 | 0 | 1 | 0 | 1 | 0 | 0 | 0 | 0 | 0 | 100% (1) |
|  | *S. aurorae* | ENV | 0 | Insect - 1 | 0 | 0 | 0 | 1 | 0 | 0 | 0 | 0 | 100% (1) |
|  | *S. candida* | ENV | 0 | 0 | 1 | 0 | 0 | 1 | 0 | 0 | 0 | 0 | 100% (1) |
|  | *S. fumea* | ENV | 0 | 0 | 1 | 0 | 1 | 0 | 0 | 0 | 0 | 0 | 100% (1) |
|  | *S. gemellus* | ENV | 0 | Mite -1 | 0 | 0 | 1 | 0 | 0 | 0 | 0 | 0 | 100% (1) |
|  | *S. globosa* | CLI | 5 | 0 | 0 | 0 | 2 | 1 | 0 | 0 | 0 | 0 | 60% (3) |
|  | *S. humicola* | ENV | 0 | 0 | 3 | 0 | 0 | 3 | 0 | 0 | 0 | 0 | 100% (3) |
|  | *S. istvo* | ENV | 0 | 0 | 4 | 0 | 0 | 4 | 0 | 0 | 0 | 0 | 100% (4) |
|  | *S. lignivora* | ENV | 0 | 0 | 5 | 0 | 4 | 1 | 0 | 0 | 0 | 0 | 100% (4) |
|  | *S. luriei* | CLI | 4 | 0 | 0 | 1 | 2 | 1 | 0 | 0 | 0 | 0 | 100% (4) |
|  | *S. mexicana* | ENV | 0 | 0 | 4 | 0 | 0 | 0 | 0 | 0 | 0 | 0 | 0% (0) |
|  | *S. pallida* | ENV | 1 | 0 | 4 | 0 | 1 | 4 | 0 | 0 | 0 | 0 | 100% (5) |
|  | *S. palmiculminata* | ENV | 0 | 0 | 1 | 0 | 0 | 1 | 0 | 0 | 0 | 0 | 100% (1) |
|  | *S. phasma* | ENV | 0 | 0 | 1 | 0 | 0 | 1 | 0 | 0 | 0 | 0 | 100% (1) |
|  | *S. protearum* | ENV | 0 | 0 | 2 | 0 | 1 | 1 | 0 | 0 | 0 | 0 | 100% (2) |
|  | *S. rapaneae* | ENV | 0 | 0 | 3 | 0 | 0 | 3 | 0 | 0 | 0 | 0 | 100% (3) |
|  | *S. schenckii* | CLI | 23 | 0 | 3 | 0 | 2 | 12 | 0 | 0 | 0 | 0 | 54% (14) |
|  | *S. splendens* | ENV | 0 | 0 | 2 | 0 | 1 | 1 | 0 | 0 | 0 | 0 | 100% (2) |
|  | *S. stylites* | ENV | 0 | 0 | 9 | 0 | 7 | 2 | 0 | 0 | 0 | 0 | 100% (9) |
|  | *S. thermara* | ENV | 0 | 0 | 1 | 0 | 0 | 1 | 0 | 0 | 0 | 0 | 100% (1) |
|  | *S. uta* | ENV | 0 | 0 | 4 | 0 | 0 | 4 | 0 | 0 | 0 | 0 | 100% (4) |
|  | *S. variecibatus* | ENV | 0 | Mite -2 | 3 | 0 | 4 | 1 | 0 | 0 | 0 | 0 | 100% (5) |
| Zambia (n= 2) | *S. protea-sedis* | ENV | 0 | 0 | 1 | 0 | 0 | 1 | 0 | 0 | 0 | 0 | 100% (1) |
|  | *S. zambiensis* | ENV | 0 | 0 | 1 | 0 | 0 | 1 | 0 | 0 | 0 | 0 | 100% (1) |
| ASIA | | | | | | | | | | | | | |
| Azerbaijan (n= 1) | *S. fusiforme* | ENV | 0 | 0 | 1 | 0 | 1 | 0 | 0 | 0 | 0 | 0 | 100% (1) |
| China (n= 580) | *S. globosa* | CLI | 575 | 0 | 5 | 0 | 10 | 0 | 0 | 0 | 0 | 0 | 2% (10) |
|  | *S. schenckii* | CLI | 4 | 0 | 0 | 1 | 0 | 0 | 0 | 0 | 0 | 0 | 25% (1) |
|  | *S. insectorum* | ENV | 0 | 0 | 1 | 0 | 0 | 0 | 0 | 0 | 0 | 0 | 0% (0) |
| India (n= 87) | *S. globosa* | CLI | 87 | 0 | 0 | 0 | 0 | 0 | 0 | 0 | 0 | 0 | 0% (0) |
| Iran (n= 18) | *S. globosa* | CLI | 6 | 0 | 4 | 0 | 0 | 0 | 0 | 0 | 0 | 0 | 0% (0) |
|  | *S. schenckii* | CLI | 8 | 0 | 0 | 0 | 0 | 0 | 0 | 0 | 0 | 0 | 0% (0) |
| Korea (n= 8) | *S. globosa* | CLI | 8 | 0 | 0 | 0 | 0 | 0 | 0 | 0 | 0 | 0 | 0% (0) |
| Japan (n= 144) | *S. globosa* | CLI | 117 | Cat - 1 | 0 | 0 | 5 | 0 | 60 | 0 | 0 | 0 | 55% (65) |
|  | *S. nigrograna* | ENV | 0 | 0 | 1 | 0 | 0 | 1 | 0 | 0 | 0 | 0 | 100% (1) |
|  | *S. nivea* | ENV | 0 | 1 | 0 | 0 | 1 | 0 | 0 | 0 | 0 | 0 | 100% (1) |
|  | *S. pallida* | ENV | 0 | 0 | 2 | 0 | 2 | 0 | 0 | 0 | 0 | 0 | 100% (2) |
|  | *S. schenckii* | CLI | 24 | 0 | 2 | 0 | 2 | 0 | 13 | 0 | 0 | 1 | 72% (16) |
| Malasya (n= 30) | *S. guttiliformis* | ENV | 0 | 0 | 1 | 0 | 0 | 1 | 0 | 0 | 0 | 0 | 100% (1) |
|  | *S. schenckii* | CLI | 4 | Cat - 25 | 0 | 0 | 0 | 0 | 0 | 0 | 0 | 0 | 0% (0) |
| Thailand (n= 27) | *S. schenckii* | CLI | 1 | Cat-26 | 0 | 0 | 1 | 0 | 0 | 0 | 0 | 0 | 3% (1) |
| OCEANIA | | | | | | | | | | | | | |
| Australia (n=2) | *S. eucalyptigena* | ENV | 0 | 0 | 1 | 0 | 1 | 0 | 0 | 0 | 0 | 0 | 100% (1) |
|  | *S. pallida* | ENV | 0 | Cat -1 | 0 | 0 | 0 | 0 | 0 | 0 | 0 | 0 | 0% (0) |
| New Zeland (n= 1) | *S. nothofagi* | ENV | 0 | 0 | 1 | 0 | 0 | 1 | 0 | 0 | 0 | 0 | 100% (1) |
| Tasmania (n= 1) | *S. humicola* | ENV | 0 | *Dasyurus viverrinus* -1 | 0 | 0 | 0 | 0 | 0 | 0 | 0 | 0 | 0% (0) |

Legend: HMN: Human; ANM: Animal; ENV: Environment. ATCC - American Type Culture Collection; CBS - Filamentous fungi and Yeast Collection; CMW - Culture Collection of Innovation Africa at the University of Pretoria; IFM - Medical Mycology Research Center, Chiba University; IHEM - BCCM/IHEM Fungi collection: Human and Animal Health; MUM - Micoteca da Universidade do Minho; IOC - Institute Oswaldo Cruz.
